# A Study on the Cell Layer Patterns of a Citrus Periclinal Chimera Reveals β‐Cryptoxanthin Regulation in Citrus Fruits

**DOI:** 10.1002/advs.202503177

**Published:** 2025-08-19

**Authors:** Chi Zhang, Kaijie Zhu, Zhehui Zhang, Huiyu Ji, Qun Wu, Lin Zhang, Fuzhi Ke, Gang Wang, Min Zhang

**Affiliations:** ^1^ National Key Laboratory for Development and Utilization of Forest Food Resources Zhejiang A&F University Hangzhou Zhejiang 311300 P. R. China; ^2^ Key Laboratory of Quality and Safety Control for Subtropical Fruit and Vegetable Ministry of Agriculture and Rural Affairs College of Horticulture Science Zhejiang A&F University Hangzhou Zhejiang 311300 P. R. China; ^3^ National Key Laboratory for Germplasm Innovation and Utilization of Horticultural Crops College of Horticulture and Forestry Sciences Huazhong Agricultural University Wuhan Hubei 430070 China; ^4^ Quzhou Academy of Agricultural and Forestry Sciences Quzhou Zhejiang 324000 P. R. China; ^5^ Zhejiang Agricultural Technology Extension Center Hangzhou Zhejiang 310020 P. R. China; ^6^ Zhejiang Citrus Research Institute Taizhou Zhejiang 318026 P. R. China; ^7^ Agriculture and Rural Bureau of Changshan County Quzhou Zhejiang 324200 P. R. China

**Keywords:** β‐cryptoxanthin, carotenoid, chimera, citrus, fruit development, graft

## Abstract

As a dietary provitamin A carotenoid, β‐cryptoxanthin is more bioavailable than common carotenoids. However, β‐cryptoxanthin is a minor carotenoid in most crops, except for certain citrus species. A chimera (OCC) is identified, which grows from the graft junction between mandarin (OOO) and grapefruit (CCC), and it exhibits a unique β‐cryptoxanthin profile. OCC is a periclinal chimera with the L1 cell layer from OOO and L2 and L3 layers from CCC. The flavedo, pulp, and segment membranes of OCC are generated from all three cell layers, but the proportions of these cell layers differ; OOO:CCC (L1:L2/L3) is ≈1:4 in the flavedo, 1:1 in the pulp, and 1:3 in the segment membrane. A nucleus‐localized transcriptional activator, *MYB107*, is identified, which regulates β‐cryptoxanthin variation between OCC and its donors. *MYB107* expression is closely associated with β‐cryptoxanthin accumulation, and its overexpression in citrus calli and fruits enhance carotenoid biosynthesis and upregulate carotenogenic genes, whereas *MYB107* expression interference demonstrates the opposite effects. *MYB107* directly binds to and activates the promoters of *β‐carotene hydroxylase* (*BCH*), positively regulating β‐cryptoxanthin biosynthesis. These findings provide new insights into β‐cryptoxanthin accumulation patterns in citrus, highlighting the potential to improve the nutritional and aesthetic value of citrus fruits.

## Graphical abstract

Researchers have identified a citrus chimera with distinctive tissue origins and carotenoid profiles. It is discovered that fruit tissues develop from all three cell layers but in different proportions. A key transcription factor, MYB107, regulates β‐cryptoxanthin production by directly activating carotenoid biosynthesis genes, explaining why some citrus fruits develop deep orange coloration while others remain lighter.

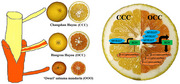

## 1 Introduction

Among fruit crops, citrus has the highest annual production worldwide (Food and Agriculture Organization of the United Nations, FAO Statistics, 2019). Citrus abounds in dietary carotenoids, which are precursors of vitamin A and other antioxidants that are indispensable for human health.^[^ ^1^ ^]^ Insufficient carotenoid intake causes vitamin A deficiency, a pressing public health problem affecting the lives of millions.^[^ ^2^ ^]^ Increasing attention is being paid to carotenoid metabolism in citrus because it has the largest repertoire of carotenoids (≈115 types) among all fruits.^[^ ^3^ ^]^ Citrus species differ widely in carotenoid composition and concentration; making it an ideal model plant for carotenoid studies. Such studies will also help breed citrus species with high carotenoid content and thus contribute to improving carotenoid sources in the human diet.

β‐Cryptoxanthin, which has been verified by observational, in vitro, animal, and human studies, can help decrease the risk of some cancers and degenerative diseases, as well as delay osteoporosis.^[^ ^4^ ^]^ β‐Cryptoxanthin is more bioavailable than most other common carotenoids.^[^ ^5^ ^]^ Recent in vivo research showed that it has a stronger effect on fat reduction and protection from oxidative stress than lycopene and β‐carotene.^[^ ^6^ ^]^ However, only a few fruits and vegetables have high β‐cryptoxanthin concentrations, with citrus fruits being the best source of dietary β‐cryptoxanthin.^[^ ^4^ ^]^ Nevertheless, only a few citrus species, mainly mandarins, can accumulate β‐cryptoxanthin predominantly in the mature fruit,^[^ ^7^ ^]^ whereas most sweet oranges predominantly accumulate violaxanthin in the fruit.^[^ ^8^ ^]^ Unfortunately, citrus species with large fruits, such as pummelos and grapefruits, contain fewer carotenoids, especially β‐cryptoxanthin.^[^ ^9^ ^]^ Further, the molecular mechanisms underlying the diversity of β‐cryptoxanthin accumulation across citrus species remain poorly understood. Because β‐cryptoxanthin accumulation does not occur in model plants such as Arabidopsis and tomato, the underlying regulatory mechanism is largely unexplored.

Chimeras, which originate from the conglomeration of cells of more than one genotype, are widely found in plants. For example, variegated leaves and flowers, some of the most common chimeras, are striking components of modern landscaping.^[^ ^10^ ^]^ In 1674, the first chimera to be described scientifically in the horticultural literature was a citrus graft chimera named “Bizzaria”, discovered from the graft junction of *Citrus aurantium* and *Citrus medica*.^[^ ^11^ ^]^ The generation of “Bizzaria” intrigued scientists but also perplexed them for centuries due to its complexity.^[^ ^10^ ^]^ Owing to heterozygosity and the long juvenile phase in some woody perennial crops such as citrus, hybridization‐based breeding is time‐ and labor‐intensive and cost‐inefficient. Thus, graft chimeras have been vital in breeding strategies in the horticultural history of *Citrus*.^[^ ^12^ ^]^ Remarkably, graft chimeras can produce excellent interspecies varieties, which are difficult to obtain by other breeding strategies; these interspecies chimeras harbor beneficial heterospecies combinations.^[^ ^13^ ^]^ Furthermore, as a graft chimera develops from different species’ cells, it is a largely under‐appreciated idea material, yet a powerful tool for studying cell lineages during development, context‐dependent gene function, intercellular communication, and heterogenomicity.^[^ ^10^ ^]^

The study of chimeras is interesting but perplexing, and the concept of the “tunica‐corpus” is key to studies on chimeras. Most higher plants have shoot apical meristems with distinct cell layers—including the outer tunica layer (L1), inner tunica layer (L2), and inner corpus (L3)—which finally develop into different plant tissues and organs, with the tunica layers dividing anticlinally and the inner corpus layers dividing both anticlinally and periclinally.^[^ ^14^ ^]^ Periclinal chimeras are ideal materials for studying how these cell layers contribute to different tissues and organs; many such chimeras have been artificially created for such studies.^[^ ^15^ ^]^ Morphological, physiological, and cytological studies have been extensively conducted in cell‐layer development models of different tissues and organs.^[^ ^16^ ^]^ However, owing to the lack of evidence from metabolic and molecular biology studies, the exact mechanisms remain unclear. Fortunately, rapid advancements in molecular biology and genome sequencing technologies are beginning to contribute to chimera research. For example, RNA‐seq in a periclinal chimeric tomato determined the layer‐specific expression of genes and uncovered the functions of different cell layers during development.^[^ ^17^ ^]^ Further, differences in DNA methylation have been observed and studied in graft chimeras.^[^ ^18^ ^]^ However, these findings mark the tip of the iceberg in the investigation of graft chimeras, and deeper molecular mechanisms remain to be elucidated.

**Figure 1:**
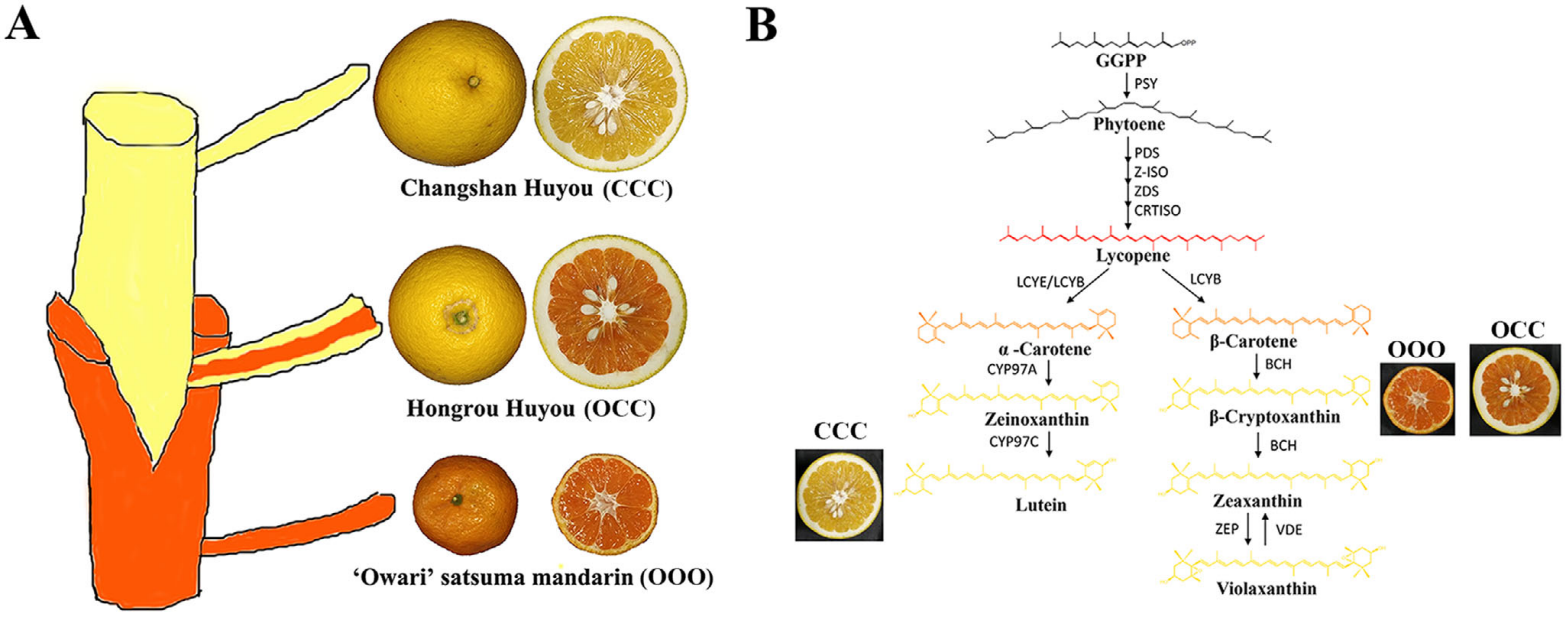
Schematic diagram of the generation of “Hongrou Huyou” (OCC) and the major carotenoid in the pulp of OCC and its donors. A) The generation of OCC. The scion “Changshan Huyou” (CCC) (upper) is grafted onto the stock “Owari” satsuma mandarin (OOO) (bottom). OCC is a graft chimera arising from the graft junction of stock and scion (middle). B) The major carotenoid in the pulp of OCC and its donors. OCC and its donors are located in the pathway side of the major carotenoid accumulating in the pulp of the deep‐colored fruit. OCC and OOO accumulate copious β‐cryptoxanthin in their pulp, but CCC only accumulates some lutein in its pulp.

Recently, a serendipitous mutation, “Hongrou Huyou” (OCC), piqued our interest. It is a periclinal chimera, initially discovered in 2001 in an orchard in Changshan County (Zhejiang Province, China), growing from the graft junction between “Owari” satsuma mandarin (OOO) and “Changshan Huyou” (CCC). CCC has light‐yellow flavedo and pulp because of low carotenoid; in contrast, OOO exhibits a deep‐orange color due to high concentrations of carotenoids, especially β‐cryptoxanthin (**Figure** **1**A). Interestingly, OCC combines desirable agronomic traits from its donors: its external phenotype is similar to that of CCC, whereas its pulp has an attractive color and is rich in β‐cryptoxanthin, similar to OOO. Thus, the chimera has large fruits with high β‐cryptoxanthin levels (Figure 1A). OCC and OOO accumulate copious β‐cryptoxanthin in the pulp, but CCC accumulates some lutein in the pulp (Figure 1B). Thus, this valuable chimera and its donors, which markedly differ in β‐cryptoxanthin accumulation, are ideal experimental materials for understanding the molecular mechanism of β‐cryptoxanthin accumulation in citrus. Metabolite profile comparison of OCC with its donors revealed the unique accumulation of metabolites in OCC.^[^ ^19^ ^]^ Simple sequence repeat (SSR) analysis found that OCC possessed nuclear, chloroplast, and mitochondrial genomes from both donors.^[^ ^20^ ^]^ However, the molecular mechanism underlying the genetic origin and β‐cryptoxanthin accumulation of OCC remain unclear. To address these unresolved aspects, we systematically studied OCC and its donors, gleaned insights on the cell‐layer development model in citrus fruits, and dissected the regulatory mechanism of β‐cryptoxanthin accumulation in different citrus species.

## 2 Results

### 2.1 OCC Simultaneously Inherits Superior Properties from Both Donors

“Hongrou Huyou” (OCC) is a spontaneous bud mutant arising from the graft junction of “Owari” satsuma mandarin (OOO) and “Changshan Huyou” (CCC) (Figure 1A). Intriguingly, the pulp differs from that of the scion; pulp color becomes abnormally red, similar to that of the stock. To characterize the temporal onset of this variation, fruits were sampled 85–195 d after flowering (DAF). The appearance of OCC flavedo resembled that of CCC (scion), in contrast to that of OCC pulp, which was similar to OOO (stock) pulp during all fruit developmental stages (Figure S1, Supporting Information).

**Figure 2:**
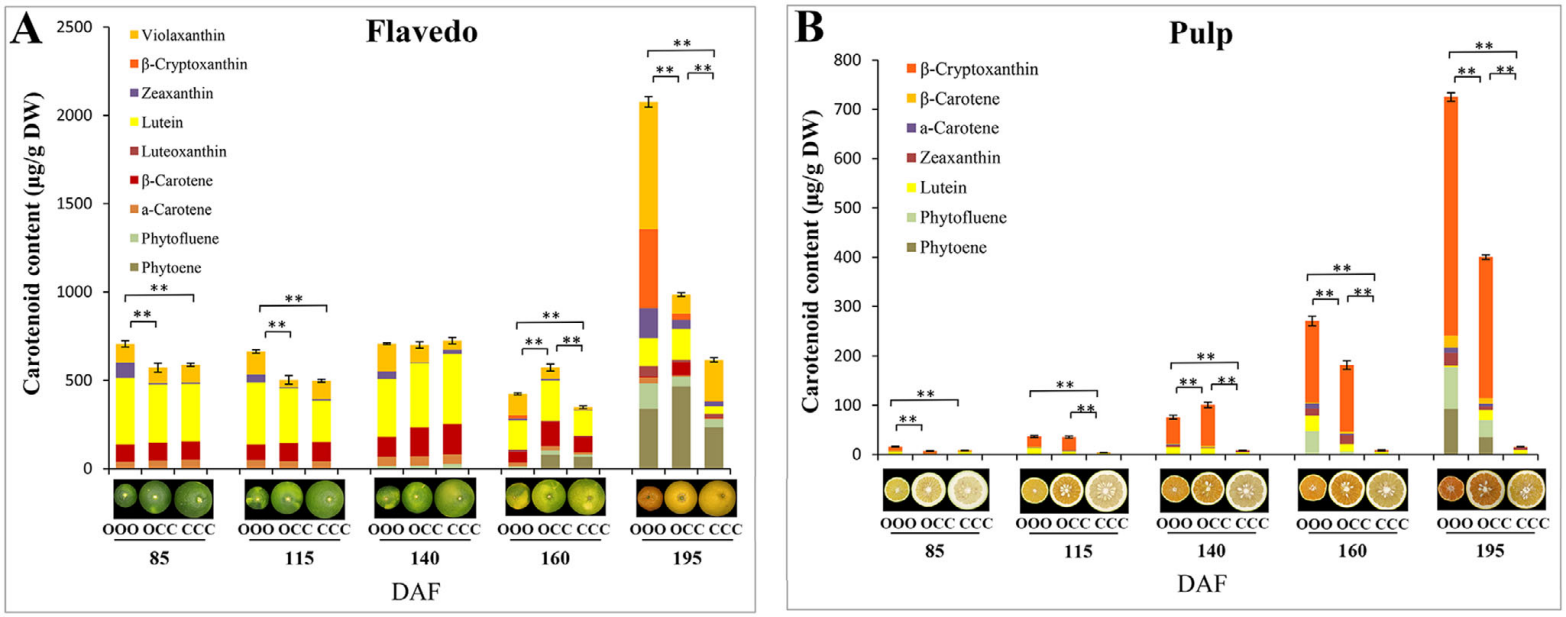
Carotenoid content of “Owari” satsuma mandarin (OOO), “Hongrou Huyou” (OCC), and “Changshan Huyou” (CCC) during fruit maturation. A) Changes in the carotenoid content in the flavedo of OCC and its donors at five developmental stages. B) Changes in the carotenoid content in the pulp of OCC and its donors at five development stages. The results are means ± SD from three biological replicates. Asterisks indicate statistically significant differences (Student's *t*‐test *P*‐value, *P < 0.05, **P < 0.01).

To understand the phenotypes of OCC, the pigments in the flavedo and pulp of OCC and its donors at different developmental stages were analyzed. In the flavedo, the total carotenoid content in OOO differed significantly from that in OCC and CCC during the first two stages (85 and 115 DAF); no differences were observed over the next 140 DAF (**Figure** **2**A and Table S1, Supporting Information). Notably, OCC had the highest carotenoid concentration at 160 DAF, in contrast to OOO at 195 DAF. Unexpectedly, β‐cryptoxanthin was also detected in the flavedo of OCC, implying that carotenoid accumulation in the flavedo of OCC differs from that of CCC despite their similar flavedo color (Figure 2A and Table S1, Supporting Information). In the pulp, the total carotenoid content of OOO differed significantly from that of OCC and CCC 85 DAF; thereafter, the total carotenoid content of OCC increased and was no different from that of OOO at 115 DAF. Remarkably, the total carotenoid content of the pulp differed significantly among these three species at 140, 160, and 195 DAF, suggesting that the carotenoid content in the pulp of OCC differs from that in OOO, although their pulp color is similar (Figure 2B and Table S2, Supporting Information). Thus, β‐cryptoxanthin is the major carotenoid in pulp of OCC and OOO (Figure 2B and Table S2, Supporting Information), and its content in OCC flavedo and pulp differs significantly from that of its donors.

### 2.2 Unique Metabolic Profiling of OCC

**Figure 3:**
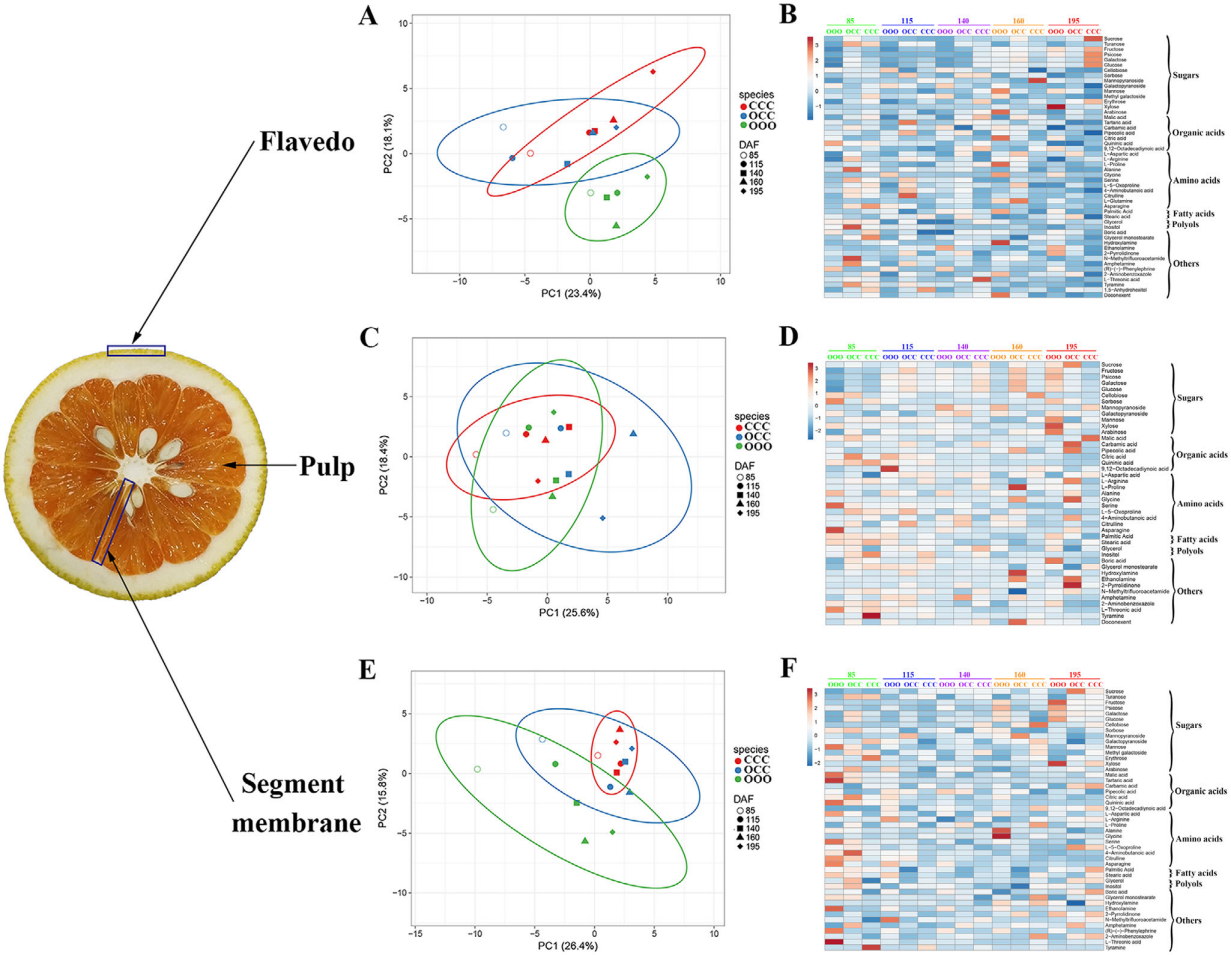
Primary metabolite compounds and concentrations among various tissues of “Owari” satsuma mandarin (OOO), “Hongrou Huyou” (OCC), and “Changshan Huyou” (CCC) during different developmental stages. A) Principal component analysis (PCA) and B) heatmap analysis of primary metabolites in the flavedo. C) PCA and D) heatmap analysis of primary metabolites in the pulp. E) PCA and F) heatmap analysis of primary metabolites in the segment membrane.

Primary metabolites, including organic acids and sugars, determine fruit quality and taste. To further study the differences between OCC and its donors, primary metabolites, volatiles, and phytohormones were analyzed. Principal component analysis (PCA) of primary metabolites in the flavedo could distinguish OOO from the others, and OCC was obtained together with CCC, indicating that OCC was similar to CCC in flavedo primary metabolites (**Figure** **3**A). Heatmap analysis also indicated that the content of most primary metabolites of OCC was between that of CCC and OOO. For example, sucrose content values in OOO, OCC, and CCC were 13 610.27, 17 190.47, and 41 181.61 µg g^−1^, respectively, during fruit ripening (195 DAF) (Figure 3B and Table S3, Supporting Information). However, the content of a few primary metabolites in OCC was significantly higher or lower than that of CCC and OOO. For instance, fructose content values in OOO, OCC, and CCC were 13 524.98, 8115.22, and 16 206.25 µg g^−1^, respectively, during fruit ripening (195 DAF); therefore, OCC fructose content was lower than that of CCC and OOO (Figure 3B and Table S3, Supporting Information). Conversely, PCA of primary metabolites in the pulp could not distinguish between these species (Figure 3C), whereas heatmap analysis showed many metabolic differences between OCC and its donors (Figure 3D and Table S4, Supporting Information). Additionally, PCA of primary metabolites in the segment membrane could not sufficiently distinguish these three species; nevertheless, OCC was closer to CCC than OOO (Figure 3E). Heatmap analysis also showed many metabolic differences between OCC and its donors (Figure 3F and Table S5, Supporting Information). The primary metabolites of OCC clustered with those of CCC in the flavedo but were hardly distinguishable in the pulp and segment membranes, and thus, OCC possesses a unique primary metabolite profile.

Volatile compounds determine the commercial value of citrus and can be clearly distinguished among various citrus species. PCA of these volatiles in the flavedo indicated that OOO was clearly distinguished from the other species, and OCC clustered with CCC (Figure S2A, Supporting Information), which means that OCC was similar to CCC for flavedo volatiles. Heatmap analysis further validated this result: compared to OOO, most volatiles were higher in OCC and CCC, especially many crucial volatiles that make distinct contributions to fruit aroma, such as d‐limonene, γ‐terpinene, and germacrene D, which are consistent with the stronger aroma of OCC and CCC (Figure S2B and Table S6, Supporting Information). However, the content of many volatiles in OCC, such as germacrene D, α‐pinene, and β‐pinene, differed sufficiently from that in CCC during fruit ripening (195 DAF). In contrast, PCA of volatiles in the pulp could not distinguish between these species (Figure S2C, Supporting Information), whereas heatmap analysis revealed many metabolite differences between OCC and its donors (Figure S2D and Table S7, Supporting Information). In addition, PCA of volatiles in the segment membrane could not distinguish these species, but the volatiles of OCC were closer to those of CCC than OOO (Figure S2E, Supporting Information). Heatmap analysis also showed metabolite differences between OCC and its donors (Figure S2F and Table S8, Supporting Information). Overall, the volatiles of OCC were similar to those of CCC in the flavedo but could not be distinguished in the pulp and segment membrane, and a unique profile of volatiles was identified in OCC.

Phytohormones, including abscisic acid (ABA), gibberellic acid (GA), indoleacetic acid (IAA), and jasmonic acid (JA), were detected in different tissues of these species across all developmental stages (Figure S3, Supporting Information). Thus, compared to its donors, OCC has a unique phytohormone accumulation pattern.

### 2.3 Generation of OCC and the Contribution of Cell Layers to Fruit Development

**Figure 4:**
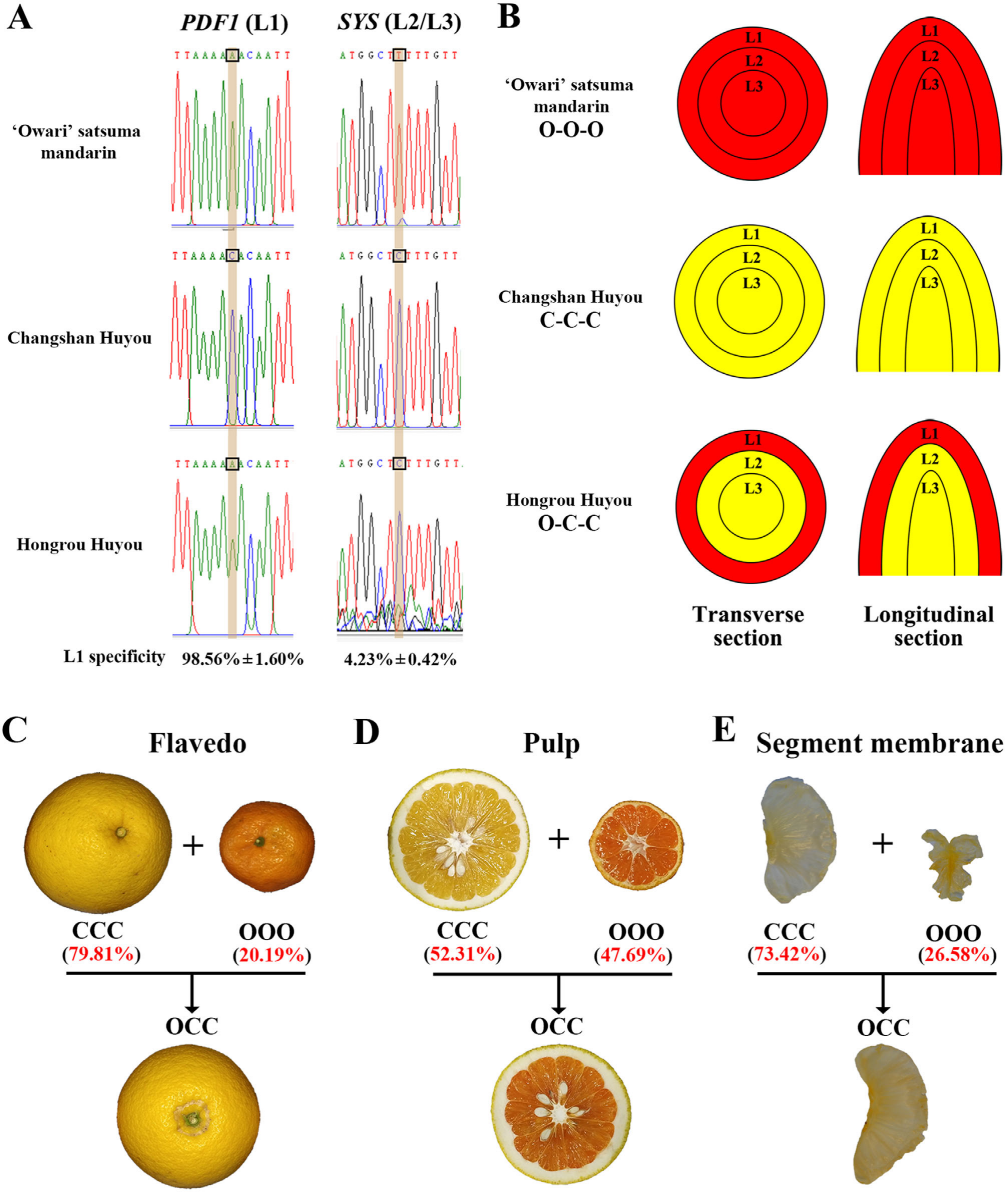
Generation of OCC and the contribution from each donor to different fruit tissues. A) Sequencing of layer‐specific expression genes. Sequencing traces of cDNA sequences of *PDF1* and *SYS* genes in OCC and its donors. Mean ± SE (Standard Error) percentage of expression in layer L1 after normalization to the amount of L1 tissue as indicated by the frequency of the OOO allele (*PDF1 n* = 3; *SYS n* = 3). B) Schematic diagrams of the cell layers in OCC and its donors. Three different cell layers are shown: the outermost L1 layer of OCC is derived from OOO, whereas the internal L2 and L3 layers of OCC are from CCC. C) Proportion from each donor in OCC flavedo. D) Proportion from each donor in OCC pulp. E) Proportion from each donor in OCC segment membrane. OOO, “Owari” satsuma mandarin; OCC, “Hongrou Huyou”; CCC, “Changshan‐Huyou”. Detailed proportions are listed in Table S9 in the Supporting Information.

To investigate chimera generation, sequence analysis of genes known to have layer‐specific expression was performed. Studies have shown that protodermal factor 1 (PDF1) has L1‐specific expression, and prosystemin (SYS) has L2/L3‐specific expression.^[^ ^17^, ^21^ ^]^ Amplification and sequencing using flavedo cDNA (complementary DNA) as a template showed that *PDF1* from OCC was identical to that from OOO, whereas *SYS* sequences from OCC were the same as those from CCC (**Figure** **4**A). The percentage expression of *PDF1* and *SYS* in L1 was also calculated, revealing L1 specificity (Figure 4A, bottom). Amplification and sequencing using the cDNA of the pulp as a template showed the same results. As its L1 layer comes from OOO and its L2/L3 layer originates from CCC, it is evident that OCC is a periclinal chimera (Figure 4B).

For a deeper understanding of the contribution of OCC donors to various tissues, we sequenced transcripts from OCC and its donors. Different SNPs between OOO and CCC were identified and SNPs (Single Nucleotide Polymorphism) in OCC were further analyzed to confirm their donor origin. Intriguingly, significant differences were found among various tissues: 79.81% of the OCC flavedo was from CCC, 52.31% of the OCC pulp came from CCC, and 73.42% of the OCC segment membrane was from CCC (Figure 4C–E and Table S9, Supporting Information). These proportions were similar across the five developmental stages (Table S9, Supporting Information). Evidently, the flavedo, pulp, and segment membranes were generated from all three cell layers, L1, L2, and L3, but the proportion of these cell layers contributing to the various tissues differed. OOO:CCC (L1:L2/L3) was ≈1:4 in the flavedo, 1:1 in the pulp, and 1:3 in the segment membrane, which explains the metabolic diversity between OCC and its donors (Figures 2 and 3 and Figure S2, Supporting Information).

KEGG (Kyoto Encyclopedia of Genes and Genomes) enrichment was performed to analyze specifically expressed genes in different layers of various tissues at 195 DAF. In the flavedo, 1001 genes originated from the L1 layer (OOO), mainly involved in glycerolipid metabolism, sulfur metabolism, and lipid biosynthesis proteins; 4847 genes came from the L2/L3 layer (CCC), mainly involved in exosome, glutathione metabolism, and ether lipid metabolism (Figure S4A, Supporting Information). In the pulp, 2145 genes originated from the L1 layer (OOO) and were mainly involved in metabolic pathways; 1850 genes came from the L2/L3 layer (CCC) and were mainly involved in signal transduction pathways (Figure S4B, Supporting Information). In the segment membrane, 1212 genes originated from the L1 layer (OOO) and were mainly involved in the secretion system, phenylalanine metabolism, and oxidative phosphorylation, and 3229 genes originated from the L2/L3 layer (CCC), being mainly involved in the ubiquitin system, ubiquitin‐mediated proteolysis, and phenylpropanoid biosynthesis (Figure S4C, Supporting Information). Thus, we identified the functional diversity of specifically expressed genes from each donor among various OCC tissues, which was closely associated with the unique metabolic profile of OCC.

### 2.4 Transcriptome Analysis of OCC and Its Donors

To decipher the molecular mechanism responsible for the alterations in OCC phenotypes, a comparative transcriptome analysis of OCC and its donors was performed. Pairwise transcriptome comparisons were performed in the flavedo, pulp, and segment membranes at the five developmental stages to identify differentially expressed genes (DEGs). In the flavedo, OCC and CCC had the fewest DEGs, implying that the flavedo of OCC is similar to that of CCC (Figure S5A, Supporting Information). Meanwhile, the number of DEGs between OCC and its donors was similar in the pulp (Figure S5B, Supporting Information). In parallel with the flavedo, the number of DEGs between OCC and CCC was the lowest in the segment membrane, implying that the segment membrane of OCC was similar to that of CCC (Figure S5C, Supporting Information). Details of these DEGs in various tissues and stages of fruit development can be seen in Figure S5D–L and Table S10–S12 in the Supporting Information. KEGG pathway (Figures S6–S8, Supporting Information), and MapMan analyses (Figure S9, Supporting Information) revealed these DEGs to be involved in various metabolic pathways, consistent with the significant differences in metabolites (carotenoids, phytohormones, primary metabolites, and volatiles) between OCC and its donors.

### 2.5 Coexpression Network Analysis

**Figure 5:**
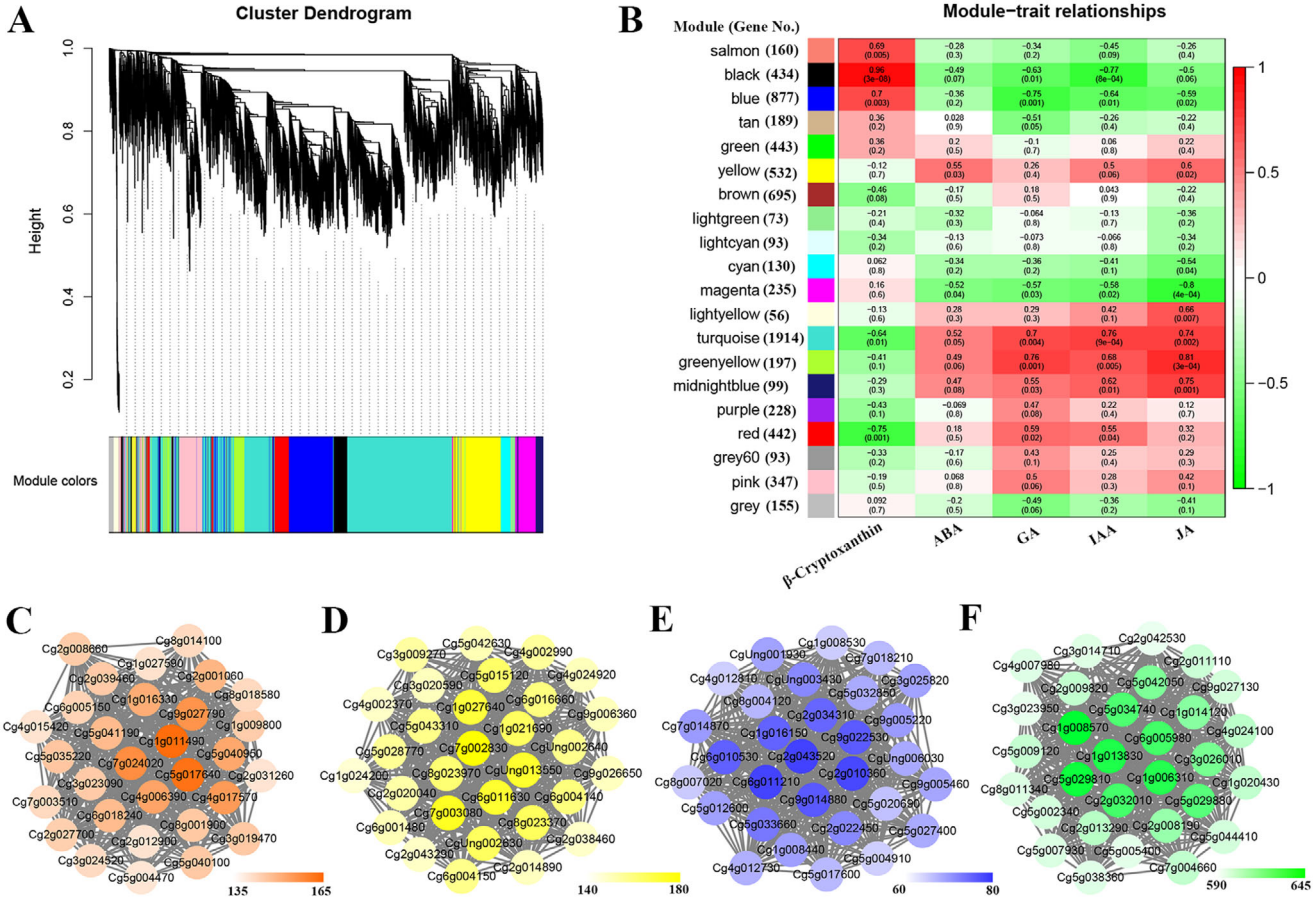
Weighted gene coexpression network analysis (WGCNA) of differentially expressed genes (DEGs) identified in the pulp of “Owari” satsuma mandarin (OOO), “Hongrou Huyou” (OCC), and “Changshan Huyou” (CCC) during different developmental stages. A) Hierarchical cluster tree of the gene network. Each of the 7392 DEGs is represented by a leaf in the tree. The major tree branches constitute 20 modules labeled in different colors. The module “Gray” is for unassigned genes. B) Module–carotenoid, ABA, GA, IAA, and JA correlations and the corresponding *P*‐values (in parentheses). Each row corresponds to a module. The left panel shows the 20 modules and the number of module member genes. Each column corresponds to a specific metabolite (carotenoid, ABA, GA, IAA, and JA). The color scale on the right shows module–trait correlation from −1 (green) to 1 (red). Cytoscape representations of the top 30 coexpressed genes with an edge weight ≥0.10 in the following modules (member gene IDs are given): C) module “Black,” related to carotenoid. The edge number of the genes ranges from 135 to 165 (color‐coded by the scale on the bottom right from white through orange). D) Module “Yellow,” related to ABA. The edge number of the genes ranges from 140 to 180 (color‐coded by the scale on the bottom right from white through yellow). E) Module “Greenyellow,” related to GA and JA. The edge number of the genes ranges from 60 to 80 (color‐coded by the scale on the bottom right from white through blue). F) Module “Turquoise,” related to IAA. The edge number of the genes ranges from 590 to 645 (color‐coded by the scale on the bottom right from white through green).

To further identify the related DEGs contributing to the above metabolite differences, weighted gene coexpression network analysis (WGCNA) was performed with the available 8864, 7392, and 7562 nonredundant DEGs identified from the flavedo, pulp, and segment membrane, respectively. In the pulp, 20 unique modules in the dendrogram were identified according to their common expression trends across all sampled tissues in the WGCNA, with each module depicted by a branch of different colors and each gene depicted by a leaf (**Figure** **5**A). β‐Cryptoxanthin, ABA, GA, IAA, and JA, were added for further analysis using these modules. Module–trait relationships showed that the “Black” module contained 434 genes that were highly correlated with β‐cryptoxanthin (*r *= 0.96, *P *= 3e^−8^), “Yellow” contained 532 genes highly correlated with ABA (*r *= 0.55, *P *= 0.03), “Greenyellow” contained 197 genes highly correlated with GA (*r *= 0.76, *P *= 0.001) and JA (*r *= 0.81, *P *= 3e^−4^), and “Turquoise” contained 1914 genes highly correlated with IAA (*r *= 0.76, *P *= 9e^−4^) (Figure 5B). The Cytoscape representation of the connections of the top 30 genes in these modules correspond with the DEGs in these modules contributing to β‐cryptoxanthin, ABA, GA, JA, and IAA (Figure 5C–F). WGCNA results for the flavedo (Figure S10, Supporting Information) and the segment membrane (Figure S11, Supporting Information) also identified modules highly correlated with these metabolites.

### 2.6 Candidate Genes Highly Associated with β‐Cryptoxanthin Accumulation

To identify candidate genes related to β‐cryptoxanthin accumulation, the “Black” module in the WGCNA of pulp was further analyzed. The 434 genes in this module were extracted and annotated (Table S13, Supporting Information). Heatmap analysis revealed that the expression patterns in OCC were similar to those in OOO (Figure S12 left, Supporting Information). Notably, the most relevant gene was the MYB family transcription factor (TF) *MYB107* (Cg1g011490). Two other candidate genes, TF *bHLH79* (Cg5g004140) and ethylene‐responsive TF *ERF112* (Cg3g018660), were also identified. *MYB107* and *ERF112* expression was positively correlated with β‐cryptoxanthin accumulation, whereas *bHLH79* expression was negatively correlated (Figure S12 right, Supporting Information). These results were confirmed by quantitative reverse transcription Polymerase Chain Reaction (RT‐qPCR) (Figure 7A and Figure S14A,B, Supporting Information).

In the flavedo, the “Green” module in the WGCNA was also analyzed to identify candidate genes involved in β‐cryptoxanthin accumulation. The 398 genes in this module were extracted and annotated (Table S14, Supporting Information). Heatmap analysis revealed that the expression patterns in OCC were similar to those in CCC (Figure S13 left, Supporting Information). Interestingly, *MYB107* was also identified. Analysis of the expression patterns of *MYB107*, TF *ERF062* (Cg9g012290), and ethylene‐responsive TF *ERF003* (Cg1g019920) revealed that these genes were positively correlated with β‐cryptoxanthin accumulation (Figure S13 right, Supporting Information). RT‐qPCR confirmed these findings (Figure 7B and Figure S14G,H, Supporting Information).

### 2.7 Genotype and Transcript Profiles of Carotenoid Pathway Genes

**Figure 6:**
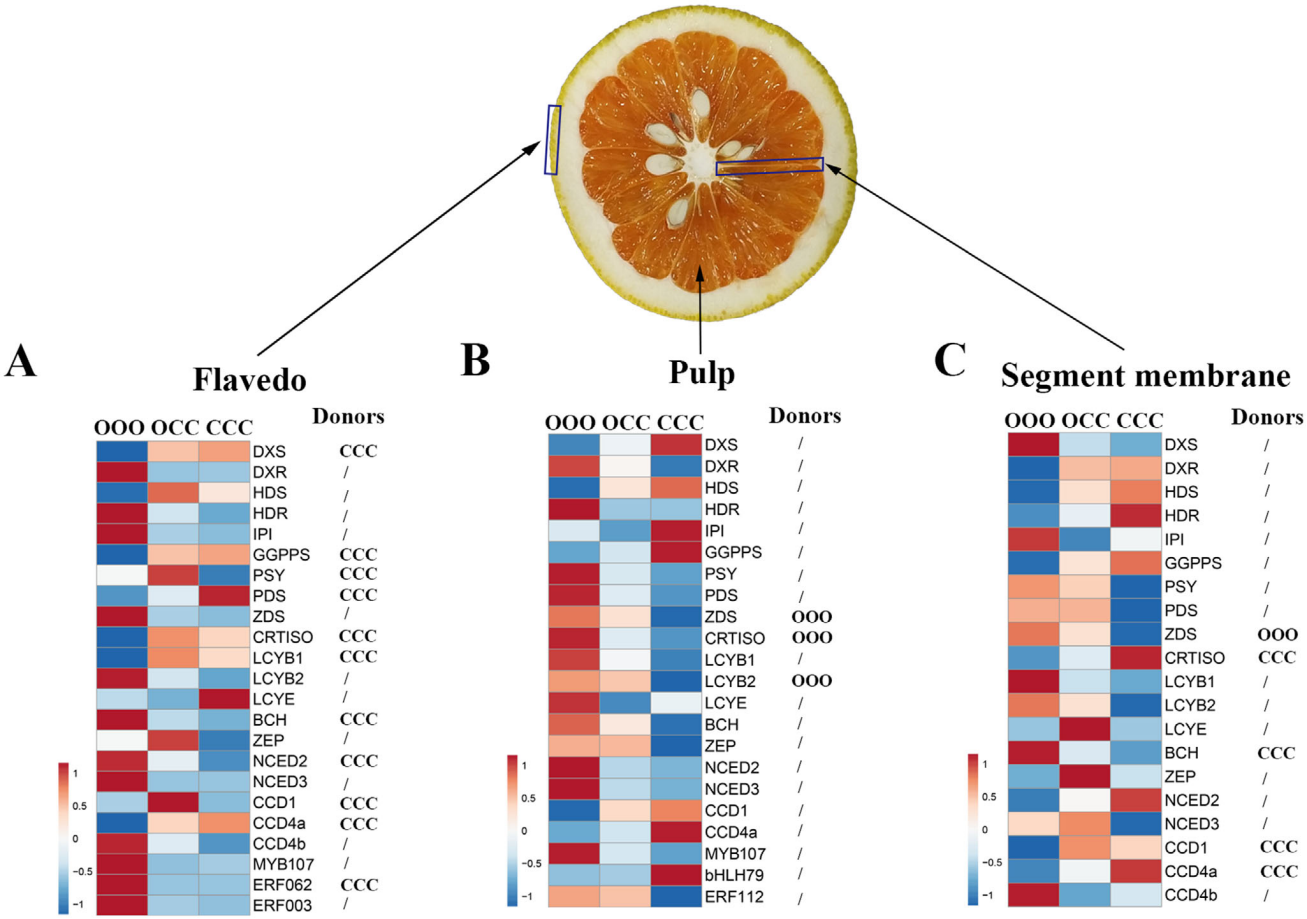
Expression patterns of carotenogenic genes identified in different tissues of “Owari” satsuma mandarin (OOO), “Hongrou Huyou” (OCC), and “Changshan Huyou” (CCC) at 195 DAF. A) Heatmap analysis of the expression of carotenogenic genes in the flavedo (left); donor origin of carotenogenic genes in OCC (right). B) Heatmap analysis of the expression of carotenogenic genes in the pulp; donor origin of carotenogenic genes in OCC (right). C) Heatmap analysis of the expression of carotenogenic genes in the segment membrane; donor origin of carotenogenic genes in OCC (right). OOO, “Owari” satsuma mandarin; OCC, “Hongrou Huyou”; CCC, “Changshan Huyou”; /, heterozygote.

To identify the expression profiles of carotenogenic genes, over 20 related genes (including the above TFs) were investigated. Heatmap analysis of the flavedo showed that the transcript levels of *DXR*, *HDR*, *IPI*, *ZDS*, *LCYB2*, *NCED2*, *NCED3*, *CCD4b*, *MYB107*, *ERF062*, and *ERF003* were significantly positively correlated with β‐cryptoxanthin accumulation, and the expression levels of these genes in OOO were dramatically higher than in OCC or CCC at 195 DAF (**Figure** **6**A). Heatmap analysis of the pulp showed the transcript levels of *DXR*, *HDR*, *PSY*, *PDS*, *ZDS*, *CRTISO*, *LCYB1*, *LCYB2*, *BCH*, *ZEP NCED2*, *NCED3*, *MYB107*, and *ERF112* to be significantly positively correlated with β‐cryptoxanthin accumulation, and the expression levels of these genes were in the following decreasing order at 195 DAF: OOO > OCC > CCC (Figure 6B). Meanwhile, the carotenogenic genes in the segment membrane showed no difference between OCC and its donors, consistent with the carotenoid deficit in the segment membrane (Figure 6C). The expression patterns of representative genes were measured by RT‐qPCR (Figure S14, Supporting Information), confirming the RNA‐seq results. The expression profiles of carotenoid pathway genes were highly associated with β‐cryptoxanthin accumulation. The donor origin of the above carotenogenic genes in OCC was also analyzed; nearly half these genes came from CCC in the flavedo; three originated from OOO in the pulp, four came from CCC, and one came from OOO in the segment membrane (Figure 6). These results were further validated by PCR‐based sequencing of four representative genes: *PSY*, *ZDS*, *LCYB2*, and *BCH*. Accordingly, the genotypes of carotenoid pathway genes are also closely associated with β‐cryptoxanthin accumulation.

Tissue‐specific expression patterns of carotenogenic genes were identified in OCC at 195 DAF (Figure S15A, Supporting Information). The transcript levels of *DXS*, *DXR*, *HDS*, *HDR*, *IPI*, and *GGPPS* were significantly higher in the flavedo of OCC, implied by the massive accumulation of the carotenoid precursor. However, the genotypes of *PSY*, *PDS*, *CRTISO*, *LCYB1*, and *BCH* originated from CCC although they had high transcript levels, thereby inhibiting β‐cryptoxanthin accumulation. In contrast, the genotypes of *PSY*, *PDS*, *CRTISO*, *LCYB1*, and *BCH* came from OOO or a heterozygote with high transcript levels in the pulp of OCC, which enhances β‐cryptoxanthin accumulation. Meanwhile, the lower transcript levels of *LCYE* and *ZEP* push the metabolic flux to β‐cryptoxanthin biosynthesis and decreased β‐cryptoxanthin degradation, which also boosts β‐cryptoxanthin accumulation. The lower transcript levels of carotenogenic genes in the OCC segment membrane were consistent with β‐cryptoxanthin deficiency. Thus, β‐cryptoxanthin accumulation is simultaneously controlled by carotenogenic gene genotype and transcript, as well as by tissue‐specific regulation (Figure S15B, Supporting Information).

### 2.8 MYB107 Functions as a Transcriptional Activator and Localizes to the Nucleus

**Figure 7:**
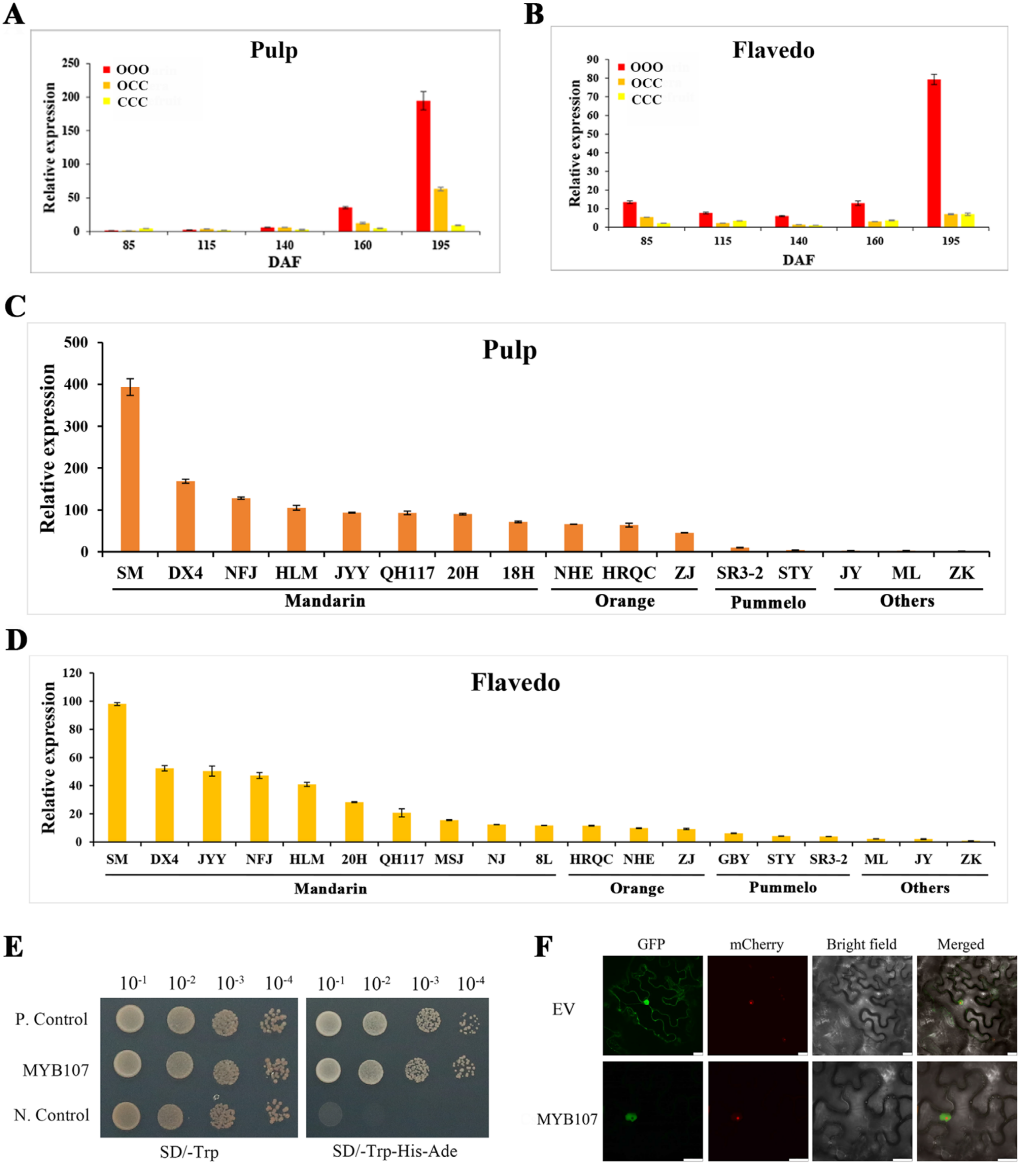
Expression levels, transcriptional activity, and subcellular localization of MYB107. Relative expression levels of *MYB107* in A) the pulp or B) flavedo in the OCC and its donors. Values are expressed as mean ± SD (*n* = 3). Relative expression levels of *MYB107* in C) the pulp or D) flavedo among various citrus species with diversity in β‐cryptoxanthin accumulation. Abbreviations are referred to Table S15 in the Supporting Information. Values are expressed as mean ± SD (*n* = 3). E) MYB107 trans‐activation activity assay. MYB107 was inserted into the pGBKT7 vector and transformed into yeast strain AH109 and the yeast cells were dotted at 10^−1^ dilutions on plates with SD/–Trp or SD/–Trp–His–Ade. P. Control, positive control (pGBKT7–53 + pGADT7‐RecT); N. Control, negative control (EV pGBKT7). Similar results were obtained in three independent biological replicates. F) Subcellular localization of MYB107 based on the visualization of the GFP signal. The fusion construct MYB107‐GFP was cotransformed with the nuclear marker gene *VirD2NLS* fused to mCherry into tobacco leaves. Confocal microscopic images of the cells were taken for GFP, mCherry fluorescence, or bright field. Bar = 20 µm. Similar results were obtained in three independent biological replicates.

To explore the expression patterns of *MYB107*, *MYB107* expression levels in the OCC and its donors were determined by RT‐qPCR. During fruit development, *MYB107* transcript levels showed a temporal increase in the pulp, and its expression levels followed the following pattern: OOO > OCC > CCC (**Figure** **7**A). Similar expression patterns were observed in the flavedo but *MYB107* transcript levels were lower (Figure 7B). In addition, we further analyzed the *MYB107* expression levels among various citrus species with diversity in β‐cryptoxanthin accumulation.^[^ ^22^ ^]^ Both RT‐qPCR and RNA‐seq results confirmed that the *MYB107* expression levels in the pulp or flavedo were higher in the citrus species with high concentration β‐cryptoxanthin (Figure 7C,D and Figure S16, Supporting Information). These results support the positive correlation of *MYB107* transcript levels with β‐cryptoxanthin accumulation, indicating that *MYB107* contributes to β‐cryptoxanthin accumulation.

Yeast 2‐hybrid analysis was performed to explore the in vivo transcriptional activity of MYB107 according to a previously described method.^[^ ^23^ ^]^ *MYB107* was inserted into the pGBKT7 vector and transformed into yeast (*Saccharomyces cerevisiae*) strain AH109. Yeast cells harboring pGBKT7‐MYB107 or the positive control (pGBKT7–53 + pGADT7‐RecT) grew on SD (Synthetic Defined Medium) /–Trp–His–Ade plates, whereas yeast cells harboring the negative control (empty vector [EV] pGBKT7) did not (Figure 7E), showing that MYB107 exhibits transcriptional activity.

To test the subcellular localization of *MYB107*, a *MYB107*‐GFP (Green Fluorescent Protein) fusion protein was constructed. MYB107‐GFP and the nuclear marker VirD2NLS‐mCherry were transiently coexpressed in tobacco leaves. GFP signals overlapped with RFP (Red Fluorescent Protein) signals, implying that the MYB107‐GFP fusion protein colocalized in vivo with the VirD2NLS‐mCherry nuclear marker in the nucleus (Figure 7F). Hence, MYB107 localizes to the nucleus.

### 2.9 MYB107 Regulates Carotenoid Biosynthesis and Carotenogenic Gene Expression in Transgenic Citrus Calli

**Figure 8:**
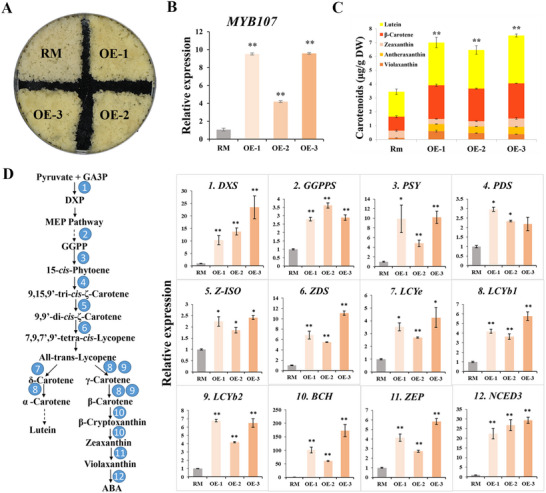
Effects of *MYB107* overexpression in citrus calli. A) Phenotypes of transgenic citrus calli. Rm, wild type; OE‐1, 2, 3, the three *MYB107*‐overexpressing citrus callus lines. B) RT‐qPCR analysis of *MYB107* transcript level. C) Carotenoid composition and content in transgenic citrus calli. D) Relative expression levels of carotenoid pathway genes in transgenic citrus calli. The genes in the carotenoid biosynthetic pathway are numbered as follows: 1, *DXS*, 1‐deoxy‐d‐xylulose 5‐phosphate synthase; 2, *GGPPS*, geranylgeranyl diphosphate synthase; 3, *PSY*, phytoene synthase; 4, *PDS*, phytoene desaturase; 5, *Z‐ISO*, 15‐*cis*‐ζ‐carotene isomerase; 6, *ZDS*, ζ‐carotene desaturase; 7, *LCYE*, lycopene *ϵ*‐cyclase; 8, *LCYb1*, lycopene *β*‐cyclase 1; 9, *LCYb2*, lycopene *β*‐cyclase 2; 10, *BCH*, *β*‐carotene hydroxylase; 11, *ZEP*, zeaxanthin epoxidase; 12, *NCED3*, 9‐*cis*‐epoxycarotenoid dioxygenase 3. DW, dry weight; GGPP, geranylgeranyl diphosphate; ABA, abscisic acid. Means ± SD from three biological replicates are shown. Asterisks indicate statistically significant differences compared with Rm (Student's *t‐*test *P‐*value, *P < 0.05, **P < 0.01).

*MYB107* was overexpressed in citrus calli to evaluate its function in citrus. Three independent transgenic lines (OE‐1, 2, and 3) were generated, which exhibited a more yellow color than the wild type (Rm) (**Figure** **8**A). RT‐qPCR results revealed that *MYB107* transcription was significantly increased in the *MYB107*‐overexpressing calli (Figure 8B). Next, we used high‐performance liquid chromatography (HPLC) to determine the carotenoid composition and content of *MYB107*‐overexpressing calli. The contents of most carotenoids, including lutein, β‐carotene, antheraxanthin, and violaxanthin, in *MYB107*‐overexpressing calli were significantly higher than those in Rm, accompanied by an increase in the total carotenoid content (Figure 8C). The transcript levels of carotenogenic genes in *MYB107*‐overexpressing calli were analyzed using RT‐qPCR, and the majority of carotenoid pathway genes, including *DXS*, *GGPPS*, *PSY*, *PDS*, *Z‐ISO*, *ZDS*, *LCYE*, *LCYb1*, *LCYb2*, *BCH*, *ZEP*, and *NCED3*, were significantly upregulated (Figure 8D), consistent with the increase in carotenoids (Figure 8C). Notably, *BCH* expression levels were dramatically upregulated in the *MYB107*‐overexpressing calli (Figure 8D). Thus, *MYB107* overexpression in citrus calli enhances carotenoid accumulation and transcription of carotenogenic genes.

### 2.10 MYB107 Positively Regulates Fruit Coloration and β‐Cryptoxanthin Accumulation in Citrus Fruits

**Figure 9:**
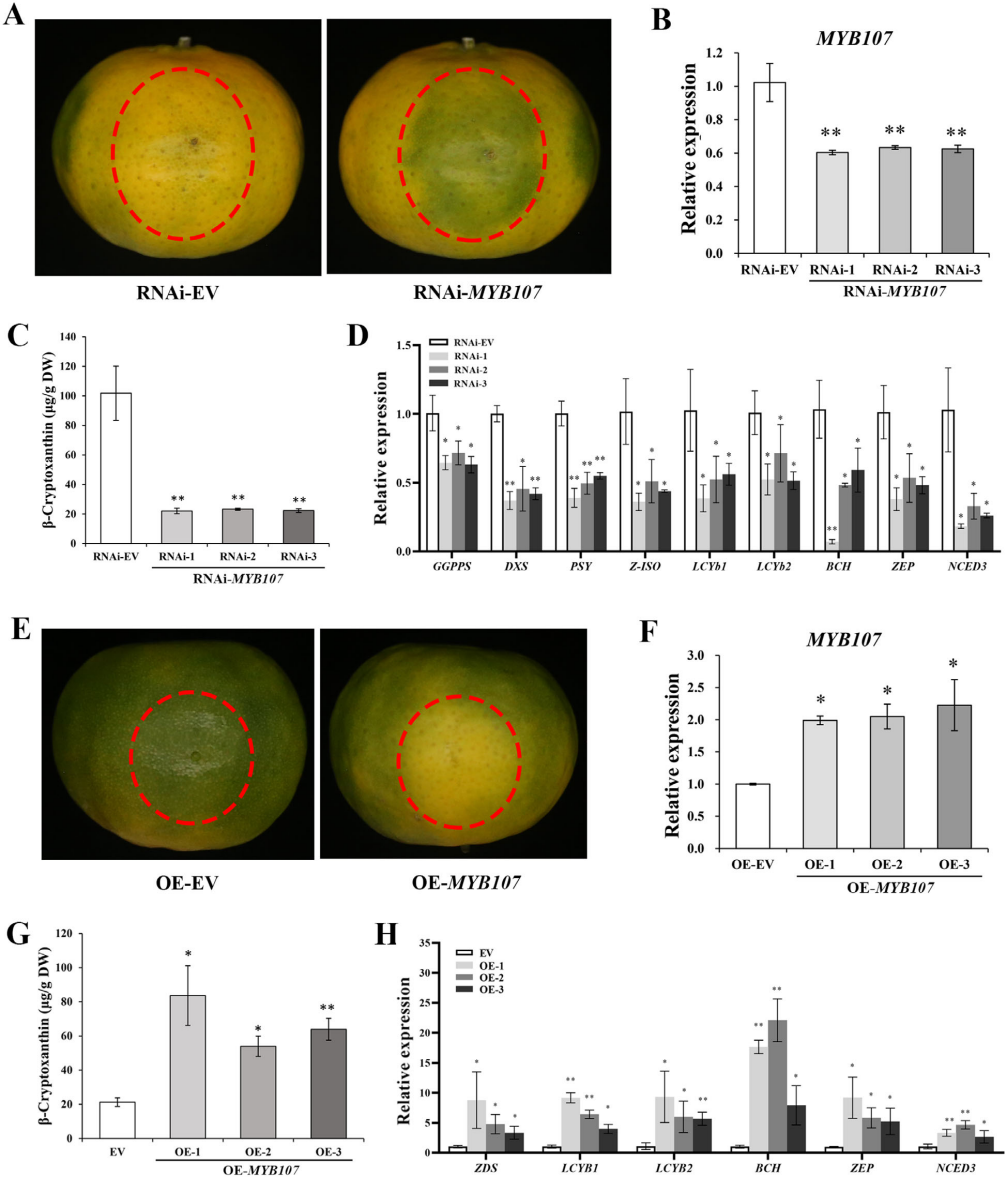
Transient interference and overexpression of *MYB107* in citrus fruits. A) Phenotype of mandarin fruits with transient interference in *MYB107* expression. RNAi‐EV, fruits injected with the RNAi empty vector (negative control); RNAi‐*MYB107*, fruits injected with the RNAi vector containing *MYB107*; RNAi‐1, RNAi‐2, and RNAi‐3, 3 RNAi‐*MYB107* lines. Similar results were obtained in three independent biological replicates. B) Relative expression of *MYB107* around the injection sites. C) β‐Cryptoxanthin content around the injection sites. D) Relative expression of carotenogenic genes around the injection sites. E) Phenotype of mandarin fruits with transient overexpression of *MYB107*. EV, fruits injected with the overexpression empty vector (negative control); OE‐*MYB107*, fruits injected with the overexpression vector containing *MYB107*; OE‐1, OE‐2, and OE‐3. Similar results were obtained in three independent biological replicates. F) Relative expression of *MYB107*. G) β‐Cryptoxanthin content. H) Relative expression of carotenogenic genes. *GGPPS*, geranylgeranyl diphosphate synthase; *DXS*, 1‐deoxy‐d‐xylulose 5‐phosphate synthase; *PSY*, phytoene synthase 1; *LCYb1*, lycopene β‐cyclase 1; *LCYb2*, lycopene β‐cyclase 2; *BCH*, β‐carotene hydroxylase; *ZEP*, zeaxanthin epoxidase; *NCED3*, 9‐*cis*‐epoxycarotenoid dioxygenase 3. Means ± SD from three biological replicates are shown. Asterisks indicate statistically significant differences compared with EV (Student's *t*‐test *P*‐value, *P < 0.05, **P < 0.01).

To confirm the function of MYB107, transient interference and overexpression experiments were performed. Mandarin fruits were injected with *Agrobacterium* carrying RNAi‐*MYB107* or an RNAi EV control. After 7 d of dark treatment, the control fruits faded from green to yellow, but RNAi‐*MYB107* fruits remained green near the injection point (**Figure** **9**A). RT‐qPCR results revealed that *MYB107* transcription significantly decreased in RNAi‐*MYB107* fruits, which implied *MYB107* expression interference (Figure 9B). β‐Cryptoxanthin content in RNAi‐*MYB107* fruits was significantly lower than that of the control (Figure 9C). Concomitantly, the expression of carotenoid biosynthesis pathway genes, including *GGPPS*, *DXS*, *PSY*, *Z‐ISO*, *LCYb1*, *LCYb2*, *BCH*, *ZEP*, and *NCED3*, reduced significantly in RNAi‐*MYB107* fruits (Figure 9D). *MYB107* expression interference significantly reduced the expression of carotenogenic genes and thus inhibited β‐cryptoxanthin accumulation. Conversely, the transient overexpression of *MYB107* accelerated fruit yellowing, enhanced β‐cryptoxanthin accumulation, and upregulated genes involved in β‐cryptoxanthin biosynthesis (Figure 9E–H). Taken together, *MYB107* positively regulates fruit coloration and β‐cryptoxanthin accumulation in citrus fruits.

### 2.11 MYB107 Directly Binds to and Activates the Promoter of *BCH*

**Figure 10:**
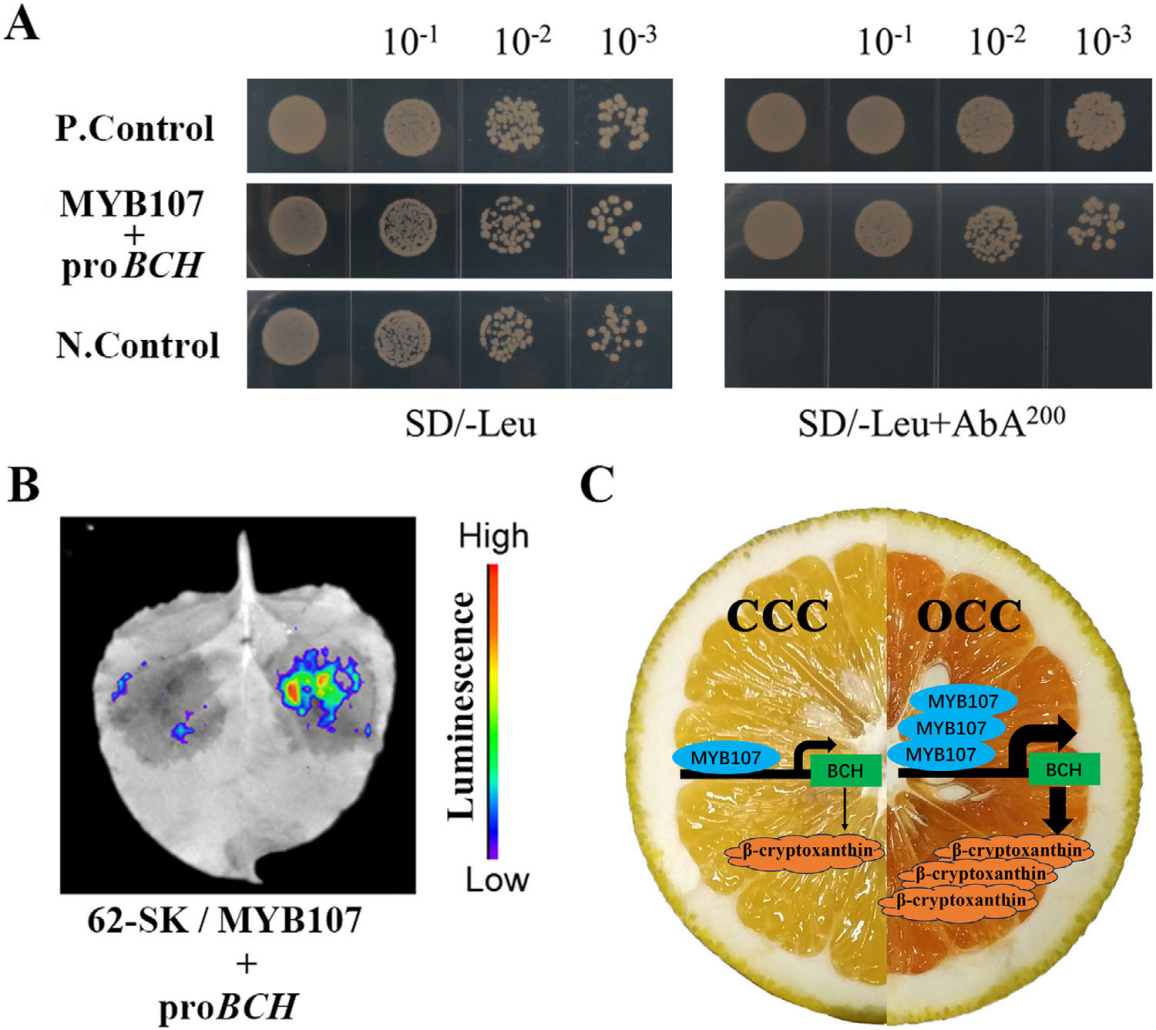
Interaction of MYB107 protein with the promoter of *BCH*. A) Y1H assay showing the binding of MYB107 to the *BCH* promoter. P. Control, positive control (pGADT7‐Rec‐p53 + p53‐AbAi); MYB107 +pro*BCH* (pGADT7‐MYB107 + pAbAi‐BCH promoter); N. Control, negative control (EV pGADT7 + pAbAi‐BCH promoter); SD/–Leu, SD medium without Leu; SD/–Leu/AbA^200^, SD medium without Leu supplemented with AbA at 200 ng mL^−1^. Transformed yeast cells were dotted at 10^−1^ dilutions on the selective medium. Similar results were obtained in three independent biological replicates. B) Dual‐LUC assay showing relative MYB107 activation to the *BCH* promoter. Luminescence imaging of tobacco leaves is shown 48 h after coinfiltration with different pairs of reporter and effector constructs. pGreenII‐62‐SK (62‐SK)+pGreenII0800‐promoter‐LUC (pro*BCH*) is the control on the left, and 62‐SK‐ MYB107+pGreenII‐0800‐promoter‐LUC (pro*BCH*) is the treatment on the right in one tobacco leaf. Similar results were obtained in three independent biological replicates. C) Working model for β‐cryptoxanthin accumulation induced by MYB107 in chimera.

To confirm the interaction between MYB107 and the promoter of *BCH*, a yeast 1‐hybrid (Y1H) assay was performed. Yeast cells were cotransformed with the positive control (pGADT7‐Rec‐p53 and p53‐AbAi), MYB107 (pGADT7‐MYB107 + pAbAi‐BCH), and negative control (EV pGADT7 + pAbAi‐BCH), and they grew well on synthetic dropout nutrient medium (SD/–Leu). However, only the yeast cells cotransformed with MYB107 and positive control survived on the selective medium supplemented with 200 ng mL^−1^ aureobasidin A (SD/–Leu/AbA^200^; **Figure** **10**A). These results indicate that MYB107 directly binds to the promoter of *BCH*. To determine whether MYB107 can activate the *BCH* promoter, we performed a dual‐luciferase assay. The *BCH* promoter was inserted into the LUC (Luciferase) reporter vector, and the coding sequence (CDS) of *MYB107* was cloned into the effector vector. The coexpression of the *MYB107* effector with the *BCH* promoter reporter dramatically increased LUC activity relative to the control (EV) (Figure 10B). Thus, MYB107 binds directly to and activates the promoters of *BCH*.

## 3 Discussion

Mutations that give rise to unusual traits in graft chimeras are a valuable source of variation, especially in slow‐cycling plants such as citrus. Such variation can play a critical role in crop improvement. In this context, a promising graft chimera, “Hongrou Huyou” (OCC), simultaneously inherits superior properties from its donors, making its fruit large, with high β‐cryptoxanthin concentrations. Besides its economic value, OCC is an important discovery for β‐cryptoxanthin research in citrus owing to its origin from CCC, a β‐cryptoxanthin‐deficient species, and OOO, a β‐cryptoxanthin‐rich species. This beneficial genetic resource might hold the key to breeding citrus varieties with high β‐cryptoxanthin accumulation. Therefore, we investigated its cell layer development and dissected the potential regulatory mechanism of β‐cryptoxanthin accumulation among various citrus species.

### 3.1 Cell‐Layer Development Model of Citrus Fruit

Graft chimeras exist in numerous plant species, including citrus. While morphological, physiological, cytological, and molecular marker studies have been conducted to evaluate species donor origin of chimeras, they have had limited success.^[^ ^12^, ^24^ ^]^ Morphological analyses of OCC and its donors have shown that the fruit size, winged leaf, seed, pollen, and rind aroma characteristics derived from L2/L3 are similar to those of CCC, whereas the juice sac and stomatal characteristics originating from L1 resemble those of OOO, implying that OCC is a periclinal chimera, with L1 from OOO and L2/L3 from CCC.^[^ ^20^ ^]^ Metabolic analysis has shown that the flavedo of OCC is derived from CCC, whereas the juice sac of OCC is derived from OOO.^[^ ^19^ ^]^ Recently, layer‐specific genes and their conserved mechanism of layer‐specific expression were found in various species.^[^ ^17^, ^21^ ^]^ Sequence analysis of these genes showed that the sequences of the L1 layer‐specific gene from OCC were identical to those from OOO, whereas the sequences of the L2/L3 layer‐specific gene from OCC were identical to those from CCC (Figure 4). Thus, OCC is a periclinal chimera, as its L1 layer originates from OOO, and its L2/L3 layer originates from CCC.

According to the “tunica‐corpus” theory, the three layers are cells of the shoot apical meristem that finally develop into different plant tissues and organs.^[^ ^14^ ^]^ How cell layers contribute to different tissues and organs is an interesting but perplexing poser that is suitable for studying through special materials, such as periclinal chimeras. Cell‐layer development models for different tissues and organs have been widely studied in plants.^[^ ^16^ ^]^ Flavedo origin from the L2 layer, pulp origin from the L1 layer, and segment membrane origin from the L2/L3 layer have been suggested.^[^ ^16^, ^24^ ^]^ However, owing to a lack of evidence from metabolic and molecular biology studies, the cell‐layer development model of citrus fruits remains unclear.

Benefitting from technological advancements, our study innovatively revealed that the flavedo, pulp, and segment membrane of OCC originate from both OOO and CCC, indicating that these fruit tissues arise from the L1 and L2/L3 cell layers (Figure 4). Accordingly, a unique metabolic profile was identified in OCC. For example, although the flavedo color of OCC is similar to that of CCC, the existence of β‐cryptoxanthin in the flavedo of OCC (Figure 2) differs dramatically from that of CCC. This confirms that the flavedo of OCC originates from both OOO and CCC, and that all tissues arise from L1 and L2/L3 layer cells. Our previous study on nuclear, chloroplast, and mitochondrial SSR analyses showed that the flavedo, juice sac, and segment membrane of OCC contains nuclear, chloroplast, and mitochondrial genomes of both donors, which also confirms that all these fruit tissues originate from both OOO and CCC.^[^ ^20^ ^]^ However, the proportion from OOO or CCC differs markedly in various tissues; OOO:CCC (L1:L2/L3) is ≈1:4 in the flavedo, 1:1 in the pulp, and 1:3 in the segment membrane. The metabolic data are consistent with these results. PCA of primary and volatile metabolites in the flavedo and segment membrane clearly showed that OCC clustered with CCC, but these metabolites in the pulp could not distinguish OCC from its donors (Figure 3 and Figure S2, Supporting Information). In conclusion, the flavedo, pulp, and segment membrane are generated from all three cell layers, albeit at varying proportions, yielding the observed diversity of metabolites between OCC and its donors.

### 3.2 Diversity in β‐Cryptoxanthin Accumulation among Various Citrus Species

We showed that the diversity in the accumulation of β‐cryptoxanthin in the flavedo and pulp between OCC and its donors is mainly regulated by the expression patterns of carotenogenic genes (Figure 6). The expression levels of carotenogenic genes, including *PSY*, *PDS*, *CRTISO*, and *LCYB1*, were higher in the flavedo of OCC than in the pulp, but the β‐cryptoxanthin content in the flavedo in OCC was much lower than that in OOO. This might be attributed to the diversity of the cell‐layer development model between the flavedo and pulp, specifically in agreement with the OOO:CCC genetic proportions of 1:4 in the flavedo and 1:1 in the pulp. Further, about half the carotenogenic genes in the flavedo of OCC are derived from CCC, whereas most carotenogenic genes in the pulp of OCC are almost heterozygous, leading to variation in the accumulation of β‐cryptoxanthin (Figure 6). Thus, the nucleotide sequence diversity of carotenogenic genes determines β‐cryptoxanthin accumulation. Carotenoid accumulation in citrus fruits has been shown to be controlled by the transcription of carotenogenic genes.^[^ ^25^ ^]^ Carotenoid composition and content vary greatly among various citrus species, being highly regulated by the transcription of carotenogenic genes.^[^ ^26^ ^]^ A possible reason for the nucleotide sequence diversity playing an important role in our study is that the genetic relationship between OOO and CCC is distant, likely causing a tremendous difference in their nucleotide sequences and resulting in the diversity of their enzyme activities. Genotypes and genetic polymorphisms have been shown to cause variation in the accumulation of carotenoids among various citrus species.^[^ ^27^ ^]^ We also analyzed the tissue‐specific expression patterns of carotenogenic genes in OCC, which further confirmed the combined effect of gene expression and genotype on β‐cryptoxanthin accumulation (Figure S15A, Supporting Information). Similarly, differences in carotenoid accumulation between leaf and fruit tissues are controlled by tissue‐specific regulation of carotenogenic genes.^[^ ^28^ ^]^ These findings lead us to suggest that β‐cryptoxanthin accumulation in OCC is collectively controlled by carotenogenic gene genotype and transcript, as well as tissue‐specific differences (Figure S15B, Supporting Information).

To systematically elucidate the regulatory mechanism of β‐cryptoxanthin metabolism in citrus fruit, we investigated 20 genes related to β‐cryptoxanthin metabolism (Figure 6), far more than those in a previous study, which included six genes.^[^ ^25a^ ^]^ We observed many interesting findings. For example, the expression level of *LCYB2*, a chromoplast‐specific lycopene β‐cyclase,^[^ ^29^ ^]^ was much higher than that of *LCYB1* (Table S16, Supporting Information), and the expression pattern of *LCYB2* had a stronger relationship with β‐cryptoxanthin accumulation. Thus, *LCYB2* rather than *LCYB1* has a crucial role in regulating β‐cryptoxanthin accumulation. Notably, the expression pattern between the flavedo and pulp was diverse: *PSY*, *ZDS*, *LCYB2*, *BCH*, and *NCED2* are critical in regulating β‐cryptoxanthin accumulation in the flavedo, but in the pulp, most carotenogenic genes were closely related to β‐cryptoxanthin accumulation (Figure 6). Contrary to most carotenogenic genes, *LCYE* expression was higher at early stages of fruit development, decreasing with maturity (Figure 6), which can help in enhancing flux into β‐cryptoxanthin biosynthesis. Consistent with this finding, the lower *LCYE* transcript levels in the pulp of OCC might also induce β‐cryptoxanthin accumulation (Figure S15A, Supporting Information). Consequently, the regulatory mechanisms underlying the expression patterns of these carotenogenic genes contribute to differences in β‐cryptoxanthin levels among various citrus species are worth to be explored in the future.

### 3.3 MYB107 Enhances β‐Cryptoxanthin Biosynthesis by Directly Targeting and Upregulating *BCH*

WGCNA identified five potential key TFs (*MYB107*, *ERF062*, *ERF003*, *ERF112*, and *bHLH79*) involved in β‐cryptoxanthin metabolism (Figure 5 and Figure S10, Supporting Information). Intriguingly, *MYB107* was double identified in both pulp and flavedo WGCNA, implying its critical regulatory role in β‐cryptoxanthin accumulation. *MYB107* was named according to a previous genome‐wide analysis of the MYB TF in citrus.^[^ ^30^ ^]^ Notably, *MYB107* was also identified as a DEG in an *orange‐pericarp* pummelo mutant,^[^ ^31^ ^]^ indicating its role in carotenoid regulation. However, its regulatory mechanism has not been explored until now. Our RT‐qPCR analysis revealed that *MYB107* transcript levels were closely associated with β‐cryptoxanthin accumulation in the OCC and its donors during fruit ripening (Figure 7). *MYB107* overexpression in citrus calli and fruits enhanced carotenoid biosynthesis and upregulated carotenogenic genes, whereas *MYB107* expression interference in citrus fruits inhibited carotenoid biosynthesis and downregulated carotenogenic genes (Figures 8 and 9). In addition, MYB107 directly binds to and activates the promoters of *BCH* and upregulates its expression, as verified by Y1H and dual‐LUC assays (Figure 10). We also explored if *MYB107* can bind to and activate the promoters of the other carotenogenic genes but the results are negative, implying *MYB107* may specifically regulate the transcription of *BCH*. Accordingly, *BCH* transcripts dramatically increased in *MYB107*‐overexpressing citrus calli and fruits but decreased in fruits with *MYB107* expression interference (Figures 8 and 9). These data demonstrate that MYB107 modulates β‐cryptoxanthin biosynthesis by directly targeting and upregulating *BCH*, which advances our understanding about the regulatory mechanism of β‐cryptoxanthin accumulation.

MYB TFs are among the largest TF families and are involved in controlling multiple developmental processes in plants.^[^ ^32^ ^]^ They also play crucial roles in regulating various secondary metabolic pathways.^[^ ^33^ ^]^ Recently, several MYB TFs have been shown to regulate carotenoid metabolism in various plant species. For instance, *SlMYB72* directly binds to *PSY*, *Z‐ISO*, and *LCYB* and regulates carotenoid biosynthesis in tomato.^[^ ^34^ ^]^ *AdMYB7* binds to and activates the promoter of *AdLCY*‐β, which contributes to carotenoid biosynthesis in kiwifruit.^[^ ^35^ ^]^ The R2R3‐MYB transcriptional factor *CrMYB68* negatively regulates *CrBCH2* and *CrNCED5* expression, thereby repressing the transformation of α‐ and β‐branch carotenoids in the flavedo of *Citrus reticulata*.^[^ ^36^ ^]^ However, MYB TFs that positively regulate carotenoid accumulation, especially β‐cryptoxanthin biosynthesis in citrus fruits, remain largely unexplored. Here, we identified and functionally characterized a key TF, *MYB107*, whose homologs in Arabidopsis and tomato are unexplored. Our study indicated that *MYB107* positively regulates β‐cryptoxanthin biosynthesis in citrus fruits and contributes to the diversity in β‐cryptoxanthin accumulation among citrus species. Notably, the transient interference and overexpression of *MYB107* in citrus fruits also affect fruit degreening (Figure 9), implying that MYB107 might be involved in chlorophyll degradation or chloro‐chromoplast transition. The functions of MYB TFs in chlorophyll degradation have been explored, with MYB44 being found to negatively regulate chlorophyll degradation by inhibiting PPH (Pheophytin Pheophorbide Hydrolase) and PAO transcription in cucumber.^[^ ^37^ ^]^ Banana MaMYB60 upregulates the expression of five chlorophyll catabolic genes by directly binding to their promoters, thus accelerating chlorophyll degradation during banana ripening.^[^ ^38^ ^]^ In citrus fruits, CrMYB33 modulates chlorophyll degradation, and carotenoid biosynthesis directly binds to and activates the promoters of two carotenogenic and chlorophyll degradation genes.^[^ ^39^ ^]^ The regulatory role of *MYB107* in chlorophyll degradation or chlorochromoplast transition merits further investigation.

Carotenoid biosynthesis is an ancient metabolic pathway widely present in plants, algae, and microorganisms. In plants, carotenoid mechanisms are essential for growth and development, contributing to photosynthesis and photoprotection, phytohormones, pollination, seed dispersal, and stress responses.^[^ ^40^ ^]^ Transcriptional regulation of carotenoid biosynthesis is vitally important and has been the subject of intensive research. Recently, an increasing number of transcriptional regulators (e.g., MADS‐box, AP2/ERF, MYB, NAC, bHLH, WRKY) involved in the carotenoid mechanism and the above functions have been identified in plant photosynthetic tissues, fruits, flowers, seeds, and roots (reviewed in Ref. [41]). Studies have also identified and explored TFs that regulate carotenoids in algae.^[^ ^42^ ^]^ For instance, UpMYB44 regulates carotenoid biosynthesis by transcriptional activation of *UpPDS* in the green tide alga *Ulva prolifera*.^[^ ^43^ ^]^ In *Chlamydomonas reinhardtii*, CrMYB1 modulates carotenoid metabolism by directly regulating several key carotenogenic genes, including *ZDS*, *CRTISO1*, *ZEP*, *VDE*, and *CHYb*.^[^ ^44^ ^]^ DbMADS negatively regulates carotenoid biosynthesis by binding to the promoters of *DbPSY* and *DbLCYb* and inhibiting their transcription in *Dunaliella* sp. FACHB‐847.^[^ ^45^ ^]^ The regulatory mechanisms of carotenoid accumulation in microorganisms have been studied but more remains to be known. *CarA* and *CarH*/*LitR* TFs are involved in light‐induced transcriptional regulation of carotenogenic genes in a wide variety of nonphototrophic bacteria.^[^ ^46^ ^]^ In fungi, a putative Zn_2_Cys_6_ fungal TF *Cmcrf1* regulates carotenoid biosynthesis in *Cordyceps militaris*.^[^ ^47^ ^]^ Overall, the roles of TFs in carotenoid biosynthesis in various systems cannot be understated.

Based on our findings, we proposed a working model for *MYB107* in β‐cryptoxanthin variation among various citrus species (Figure 10C). In OCC, *MYB107* directly binds to and activates the promoter of *BCH*, upregulating the transcript levels of *BCH*, which finally enhances β‐cryptoxanthin biosynthesis, causing the deep‐orange colored pulp of OCC. In CCC, low *MYB107* expression results in decreased *BCH* transcripts, leading to the light‐yellow color in CCC pulp because of β‐cryptoxanthin deficit. We clearly demonstrated that *MYB107* positively regulates β‐cryptoxanthin biosynthesis, which might be an important factor causing variation in the accumulation of β‐cryptoxanthin among various citrus species (Figure 10C). *MYB107* could be a key target for improving fruit color and nutrition in citrus and even other horticultural crops.

In conclusion, an excellent graft chimera mutant, “Hongrou Huyou”, and its donors were systematically studied, which revealed that the flavedo, pulp, and segment membrane are generated from all three cell layers, L1, L2, and L3, but the proportion that these cell layers from both donors contribute to the different tissues varies considerably. We further uncovered that carotenogenic gene genotypes and transcripts, as well as tissue‐specific regulation, collectively control the diverse accumulation patterns of β‐cryptoxanthin in different citrus species. Notably, a critical TF, *MYB107*, was found to modulate β‐cryptoxanthin biosynthesis by directly binding to and activating the promoter of *BCH*. These findings shed new light on the cell‐layer development model of citrus fruits and decipher the potential regulatory mechanism of β‐cryptoxanthin accumulation among various citrus species. In future studies, the four other key TFs (*ERF062*, *ERF003*, *ERF112*, and *bHLH79*) should be explored for their roles in the regulation of β‐cryptoxanthin metabolism in citrus fruit. This will augment breeding efforts that aim to improve the nutritional and aesthetic value of citrus, and perhaps other fruit and vegetable crops.

## 4 Experimental Section

### Plant Materials

“Hongrou Huyou” (OCC) (*Citrus unshiu* + *Citrus changshan‐huyou*) is a bud mutant arising from the graft junction of interstock “Owari” satsuma mandarin (OOO) (*Citrus unshiu*) and scion “Changshan Huyou” (CCC) (*Citrus changshan‐huyou*). OCC and its donors were separately propagated by grafting (with *Poncirus trifoliata* as the rootstock) in 2005. The OCC mutant phenotype was stable from 2007 to 2024. OCC trees and their donors were cultivated side‐by‐side. Fruit samples were collected at five different developmental stages, from immature green to fully colored: 85, 115, 140, 160, and 195 DAF. The fruits of each genotype at each stage were collected from three different trees (treated as three biological replicates), with 30 representative fruits from each tree. Flavedo sampling is performed by using a scalpel to finely separate the exocarp (flavedo), avoiding penetrating the mesocarp (albedo). The flavedo, pulp, and segment membrane were separated from the sampled fruits, immediately frozen in liquid nitrogen, and kept at −80 °C until analysis.

### Carotenoid Extraction, Detection, and Analysis

For carotenoid content analysis, the samples were ground into a powder after lyophilization with a lyophilizer (LABCONCO FreeZone, Kansas City, MO, USA). Carotenoid extraction, detection, and analysis were performed using HPLC according to a citrus‐specific method.^[^ ^48^ ^]^ Carotenoids were identified based on their characteristic absorption spectra and by comparing their typical retention times with those of the standards. Peak areas were recorded at 286, 348, and 450 nm for phytoene, phytofluene, and other compounds, respectively. Carotenoid levels were quantified using calibration curves prepared with appropriate standards. At least three independent extractions were performed for each sample.

### Extraction and Analysis of Primary Metabolites

Primary metabolites were extracted and analyzed using GC‐MS (Gas Chromatography‐Mass Spectrometry) as previously described.^[^ ^49^ ^]^ In brief, fresh tissue (0.3 g) was homogenized in 2.7 mL methanol, to which 0.2 mg mL^−1^ ribitol in water (300 µL, as an internal standard) was added. The extract was ultrasonically extracted at 4 °C for 1 h at low frequency (20 kHz power, 70 W), incubated at 70 °C for 15 min, and cooled at −20 °C. The extract was centrifuged at 4000 *×* *g* for 15 min at 4 °C, and the sediments were re‐extracted once; 100 µL supernatant was dried with nitrogen gas. The extract was redissolved with 80 µL methoxyamine hydrochloride in pyridine (20 mg mL^−1^) for 90 min at 37 °C, and 80 µL BSTFA (N,O‐Bis(trimethylsilyl)trifluoroacetamide) was added and incubated at 37 °C for 30 min. Each sample was analyzed by GC‐MS (Thermo Fisher, ISQII, Waltham, MA, USA) with an FID (Flame Ionization Detector) and an Agilent TR‐5 MS capillary column (30 m × 25 µm i.d. × 0.1 µm). The oven and column temperature programs and metabolite identification and annotation were as described previously.^[^ ^49^ ^]^

### Extraction and Analysis of Volatiles

Volatiles were extracted and analyzed using GC‐MS as previously described.^[^ ^50^ ^]^ In brief, 1 g of fresh tissue was homogenized in 500 µL ultrapure water and mixed with 500 µL MTBE (Methyl tert‐butyl ether) containing 43.75 µg mL^−1^ methyl nonanoate as the internal standard. Ultrasonic extraction at 4 °C for 1 h was followed by centrifugation at 12000 *×g* for 10 min below 4 °C and filtration through a 0.22 µm membrane before GC‐MS analysis. The profiles of volatiles were analyzed by a TRACE GC Ultra GC coupled with a DSQII mass spectrometer (Thermo Fisher Scientific, Waltham, MA, USA) with a TRACE TR‐5 MS column (30 m × 0.25 mm × 0.25 µm; Thermo Scientific, Bellefonte, PA, USA). Helium was used as the carrier gas at 1 mL min^−1^ with a split ratio of 50:1 for the flavedo samples and 1:1 for the pulp samples. The parameters for the GC‐MS of the volatiles were set according to a published study.^[^ ^50^ ^]^

### Phytohormone Measurement

Phytohormones (ABA, GA, IAA, and JA) were extracted and analyzed as previously described.^[^ ^51^ ^]^

### Total RNA Isolation, cDNA Synthesis, and Reverse Transcription Quantitative PCR Analysis

Total RNA was extracted using the RN38‐EASY RNA extraction kit (Aidlab Biotechnology, Beijing, China) according to the manufacturer's instructions. Micro‐spectrophotometry and agarose gel electrophoresis were used to determine RNA concentration, integrity, and purity. First‐strand cDNA was synthesized from 1 µg of total RNA using the HiScript II Q RT SuperMix for qPCR (+gDNA wiper) (Vazyme). Gene‐specific primers used in RT‐qPCR (Table S17, Supporting Information) were designed using Primer Premier 5 software. RT‐qPCR was performed using a Roche LightCycler 480 system with 2X LightCycler 480 SYBR Green Master Mix (Roche). For each sample, quantification was performed in triplicate. *CsActin* was used as an internal control. Reaction specificity was confirmed by negative control and melting temperature calling analysis. The data were analyzed using LightCycler 480 software 1.5.0 (Roche).

### Sequence Analysis and Polymorphism Detection

Amplification primers of layer‐specific expression genes (*PDF1* and *SYS*) were designed using Primer Premier 5 software (Table S17, Supporting Information). PCR‐based sequencing analysis was performed. BioEdit (version 7.2.5) was used to align the sequences and identify polymorphisms (SNPs and indels) between species. The percentage expression in layer L1 was calculated according to a previous study.^[^ ^17^ ^]^

### RNA‐seq Library Construction, Sequencing, and Analysis

Fifteen groups of flavedo, pulp, and segment membrane samples from OCC and their donors were sequenced at five developmental stages. For each group, three independent biological replicates were subjected to RNA‐seq analysis. RNA library construction and sequencing were conducted by Novogene Bioinformatics Technology (Wuhan, China) using Illumina HiSeq 4000. Paired‐end sequencing data were further quality‐filtered with fastp (version 0.19.7) to remove pairs containing low‐quality bases (QV < 10) at a proportion of 20% or higher; paired reads were discarded if either one read contained adapter contamination, if more than 10% of bases were uncertain in either read, or if the proportion of low‐quality bases (Phred quality <5) was over 50% in either read. HISAT2 was used to map RNA‐seq data to the reference genome.^[^ ^52^ ^]^ SAMtools was used to generate an alignment file in a binary alignment map (BAM).^[^ ^53^ ^]^ Cuffdiff2 was used to identify DEGs.^[^ ^54^ ^]^ GO (Gene Ontology) and KEGG enrichment analyses were performed using TBtools.^[^ ^55^ ^]^ Hierarchical cluster and heatmap analyses were performed using ClustVis (https://biit.cs.ut.ee/clustvis/). Diagrams of the metabolic pathways were constructed using MapMan.^[^ ^56^ ^]^ Raw data were deposited in the NCBI (National Center for Biotechnology Information) database under accession number PRJNA573473.

### SNP Analysis

Quality‐filtered RNA‐seq data of OCC and its donors (OOO and CCC) were mapped to the reference genome using HISAT2.^[^ ^52^ ^]^ SAM tools were used to generate an alignment file in a BAM.^[^ ^53^ ^]^ Picard tools (http://picard.sourceforge.net) were used to create duplicates, and the Genome Analysis Toolkit (GATK, version 3.8) was employed for SNP calling of OOO and CCC.^[^ ^57^ ^]^ Different SNPs between OOO and CCC were identified, and the type of SNPs in OCC was further analyzed to confirm their donor origin using Linux. The details of these methods have been previously described.^[^ ^17^, ^58^ ^]^ The number of SNPs and the proportions from each donor in different OCC tissues were calculated (Table S9, Supporting Information). Genes corresponding to SNPs of donor origins of different species were found and further overlapped to obtain layer‐specific expression genes.

### Weighted Gene Coexpression Network Analysis

Overall, 8864, 7392, and 7562 DEGs from the flavedo, pulp, and segment membranes, respectively, were used for WGCNA, which was performed as described previously.^[^ ^59^ ^]^ WGCNA network construction and module detection were conducted using an unsigned type of topological overlap matrix; power β of 14, 12, and 9 for flavedo, pulp, and segment membrane, respectively; minimal module size of 30; and a branch merge cut height of 0.15 in the three tissues. The eigengene value was calculated for each module and used to test for associations with carotenoid, ABA, GA, JA, and IAA contents in the 30 samples. The networks were visualized using Cytoscape 3.1 (http://cytoscape.org/).

### Transcriptional Activation Analysis in Yeast Cells

For the *trans*‐activation activity assay, the CDS of *MYB107* was cloned into the pGBKT7 vector (Clontech, Palo Alto, CA, USA) to generate the recombinant vector pGBKT7‐*MYB107*. Primer sequences are listed in Table S17 in the Supporting Information. The recombinant vectors, positive control (pGBKT7–53 + pGADT7‐RecT), pGBKT7‐*MYB107*, and negative control (EV pGBKT7) were introduced into the yeast strain AH109 (Clontech). The transformed cells were spotted on SD/–Trp and SD/–Trp–His–Ade medium and incubated for 3–7 d at 30 °C. The *trans*‐activation activity was evaluated according to the growth status. The method followed a published study.^[^ ^23^ ^]^

### Subcellular Localization

The CDS of *MYB107* without the stop codon was amplified and inserted into the pRI101 vector fused with GFP under the control of the CaMV 35S promoter. The recombinant plasmid pRI101‐*MYB107*‐GFP was transformed into *Agrobacterium tumefaciens* strain GV3101 and infiltrated into tobacco leaves along with the nuclear marker 35S:VirD2NLS‐mCherry. GFP fluorescence was detected using a confocal laser scanning microscope (Leica TCS SP8, Wetzlar, Germany) with the following parameters: laser, 488 nm; intensity, 4.9%; collection bandwidth, 495–539 nm; and gain, 800. RFP fluorescence was detected using the following parameters: laser, 552 nm; intensity, 4.9%; collection bandwidth, 560–610 nm; gain, 735.

### Citrus Callus Transformation and Citrus Fruit Transient Transformation

*MYB107* CDS was cloned into the overexpression vector pH7WG2D using a Gateway Cloning System (Invitrogen). Primer sequences are listed in Table S17 in the Supporting Information. The recombinant plasmid pH7WG2D‐*MYB107* was transformed into *A. tumefaciens* strain EHA105. The citrus callus was derived from Marsh grapefruit (*Citrus paradisi* Macf.). The transformation methods for citrus callus and its growth conditions were according to an earlier study.^[^ ^60^ ^]^

*MYB107* CDS was cloned into the overexpression vector pH7WG2D, and a specific fragment of *MYB107* was cloned into the RNAi vector pK7GWIWG2D using a Gateway Cloning System (Invitrogen). The EV pH7WG2D or pK7GWIWG2D was used as the negative control. GFP was used as a reporter for the detection of transgene expression in explants. Transient transformation for citrus fruits followed a previous study.^[^ ^61^ ^]^

### Yeast 1‐Hybrid Analysis

The Y1H assay was performed using a Matchmaker Gold Yeast One‐Hybrid Library Screening System (Clontech). The *BCH* promoter was cloned into the pAbAi vector to produce the bait construct pAbAi‐pro*BCH*, and *MYB107* CDS was fused to GAL4 AD in the pGADT7 vector to generate the prey construct pGADT7‐*MYB107*. Primer sequences are listed in Table S17 in the Supporting Information. The detailed methods for the Y1H assay have been described previously.^[^ ^62^ ^]^

### Dual‐LUC Transient Expression Assay

The promoter sequence of *BCH* was cloned and inserted upstream of LUC in pGreen0800‐LUC to yield a pro*BCH*‐LUC reporter vector. The CDS of *MYB107* was cloned into the pGreenII‐62‐SK vector to obtain effector constructs. The reporter and effector constructs, together with the pSoup19 vector, were transformed into GV3101 cells. Cell suspensions containing effector or reporter constructs were mixed equally and coinfiltrated into tobacco leaves. After 2 d, LUC activity was measured using the VivoGlo Luciferin In Vivo Grade Kit (Promega, Madison, WI, USA) and imaged using a Night SHADE LB985 system (Berthold Technologies, Stuttgart, Germany).

### Statistical Analysis

All data are means ± SD of three biological replicates. Statistical analyses were conducted using Student's *t*‐test and one‐way ANOVA (Analysis of Variance) with Microsoft Excel (Microsoft Office 2021) to assess significance at *P* < 0.05 and 0.01.

### Accession Numbers

Sequence data from this article can be found in the NCBI database: MYB107 (XP_006470870), ERF062 (XP_006493082), ERF003 (XP_006465255), bHLH79 (XP_006476705), ERF112 (XP_006473086). The accession numbers of the other genes in the PCR‐based sequencing and RT‐qPCR analysis are provided in Table S17 in the Supporting Information.

## Conflict of Interest

The authors declare no conflict of interest.

## Author Contributions

C.Z. and K.Z. contributed equally to this work. M.Z. and C.Z. conceived and coordinated this project. M.Z., C.Z., K.Z., Z.Z., H.J., Q.W., L.Z., and F.K. designed and performed the experiments. Q.W., L.Z., F.K. and G.W. collected citrus tissue samples and assisted with field management. C.Z., K.Z., and M.Z. wrote the article. M.Z. and K.Z. agree to serve as the author responsible for contact and ensures communication.

## Supporting information



Supporting Information

Supplemental Table 1‐17

## Data Availability

The data that support the findings of this study are available in the Supporting Information of this article.
