# Supplementary material for: A Study on the Cell Layer Patterns of a Citrus Periclinal Chimera Reveals β‐Cryptoxanthin Regulation in Citrus Fruits
Source: Adv Sci (Weinh). 2025 Aug 19;12(41):e03177. doi: 10.1002/advs.202503177 (PMC12591197; doi:10.1002/advs.202503177)
Supplement: Supplementary file 1 — Supporting Information [file ADVS-12-e03177-s001.pdf]

Supporting Information

A Study on the Cell Layer Patterns of a Citrus Periclinal Chimera Reveals β-Cryptoxanthin Regulation in Citrus Fruits

Chi Zhang[1,2,#], Kaijie Zhu[3,#,], Zhehui Zhang[3], Huiyu Ji[3], Qun Wu[4], Lin Zhang[5], Fuzhi Ke[6], Gang Wang[7], Xiuxin Deng[3], Min Zhang[1,*]*

[1]National Key Laboratory for Development and Utilization of Forest Food Resources, Zhejiang A&F University, Hangzhou, Zhejiang 311300, PR China

[2]Key Laboratory of Quality and Safety Control for Subtropical Fruit and Vegetable, Ministry of Agriculture and Rural Affairs, College of Horticulture Science, Zhejiang A&F University, Hangzhou, Zhejiang 311300, PR China

[3]National Key Laboratory for Germplasm Innovation & Utilization of Horticultural Crops, College of Horticulture and Forestry Sciences, Huazhong Agricultural University, Wuhan, Hubei 430070, China.

[4]Quzhou Academy of Agricultural and Forestry Sciences, Quzhou, Zhejiang 324000, PR China

[5]Zhejiang Agricultural Technology Extension Center, Hangzhou, Zhejiang 310020, PR China

[6]Zhejiang Citrus Research Institute, Taizhou, Zhejiang 318020, PR China

[7]Agriculture and Rural Bureau of Changshan County, Quzhou, Zhejiang 324299, PR China

[#]These two authors contributed equally to this work.

**Corresponding author:*

Dr. Min Zhang
National Key Laboratory for Development and Utilization of Forest Food Resources
Zhejiang A&F University, Hangzhou 311300, PR China
Email: mzhang@zafu.edu.cn

Dr. Kaijie Zhu
National Key Laboratory for Germplasm Innovation & Utilization of Horticultural Crops, College of Horticulture and Forestry Sciences, Huazhong Agricultural University, Wuhan, Hubei 430070, China
Email: zhukaijie@mail.hzau.edu.cn

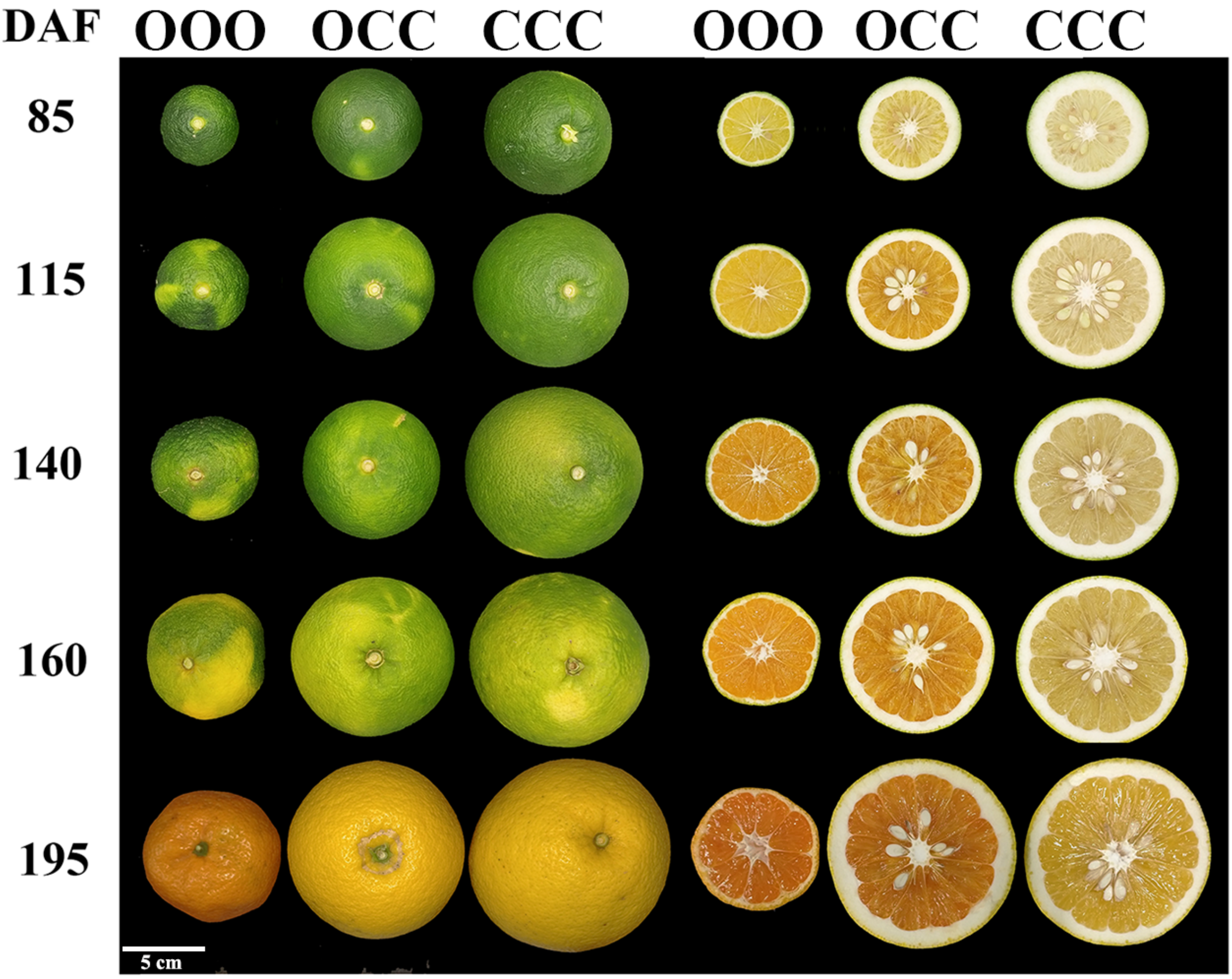

Supplementary Fig. S1 The phenotype of 'Owari' Satsuma mandarin (OOO), 'Hongrou Huyou' (OCC), and 'Changshan Huyou' (CCC) during fruit development. Phenotypes of flavedo and pulp of OCC and its donors at five development stages. Bar = 5 cm.

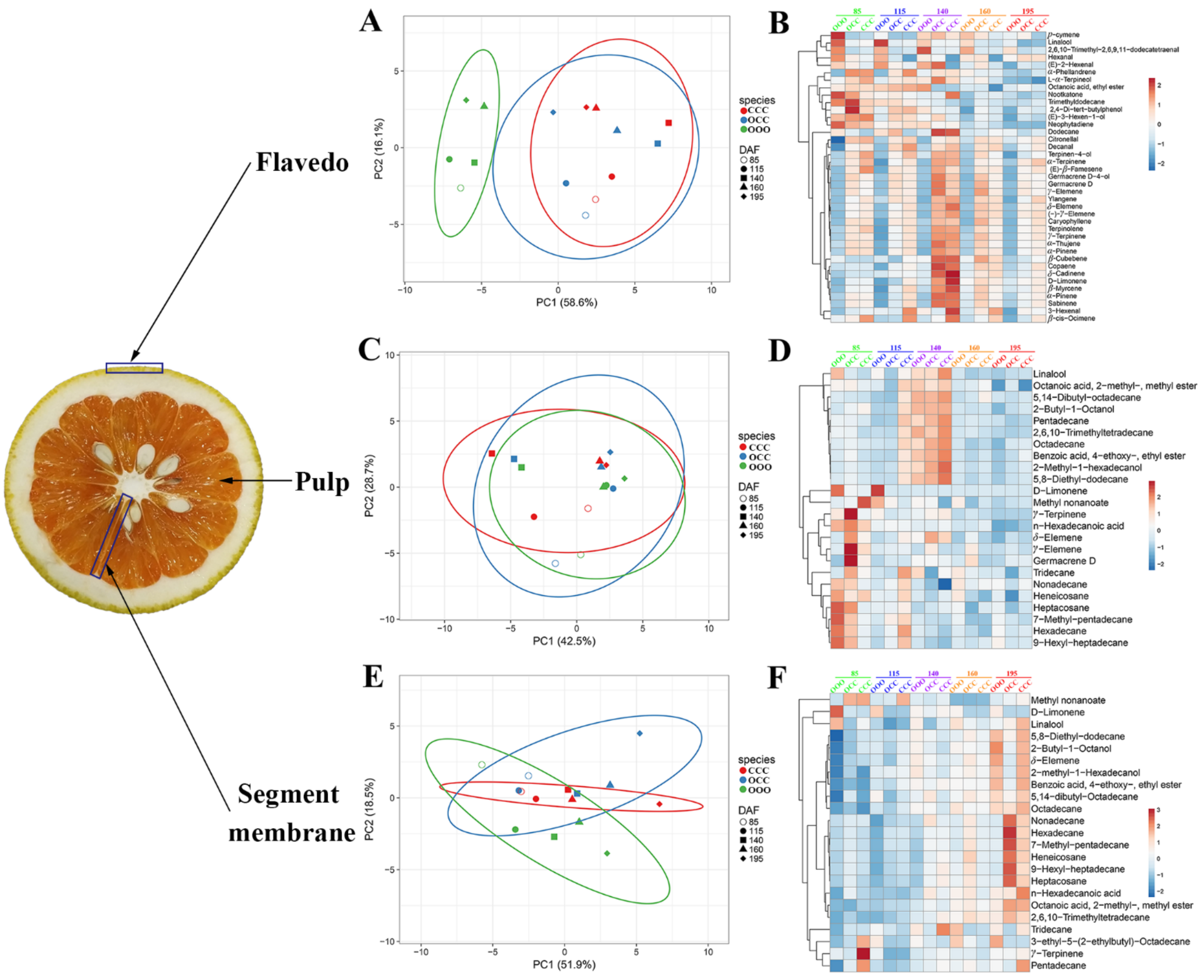

Supplementary Fig. S2 The volatile compounds and concentrations among various tissues of 'Owari' Satsuma mandarin (O), 'Hongrou Huyou' (OCC), and 'Changshan Huyou' (CCC) during different developmental stages. (A) Principal component analysis (PCA) of volatiles in flavedo. (B) Heatmap analysis of volatiles in flavedo. (C) PCA of volatiles in pulp. (D) Heatmap analysis of volatiles in pulp. (E) PCA of volatiles in segment membrane. (F) Heatmap analysis of volatiles in segment membrane.

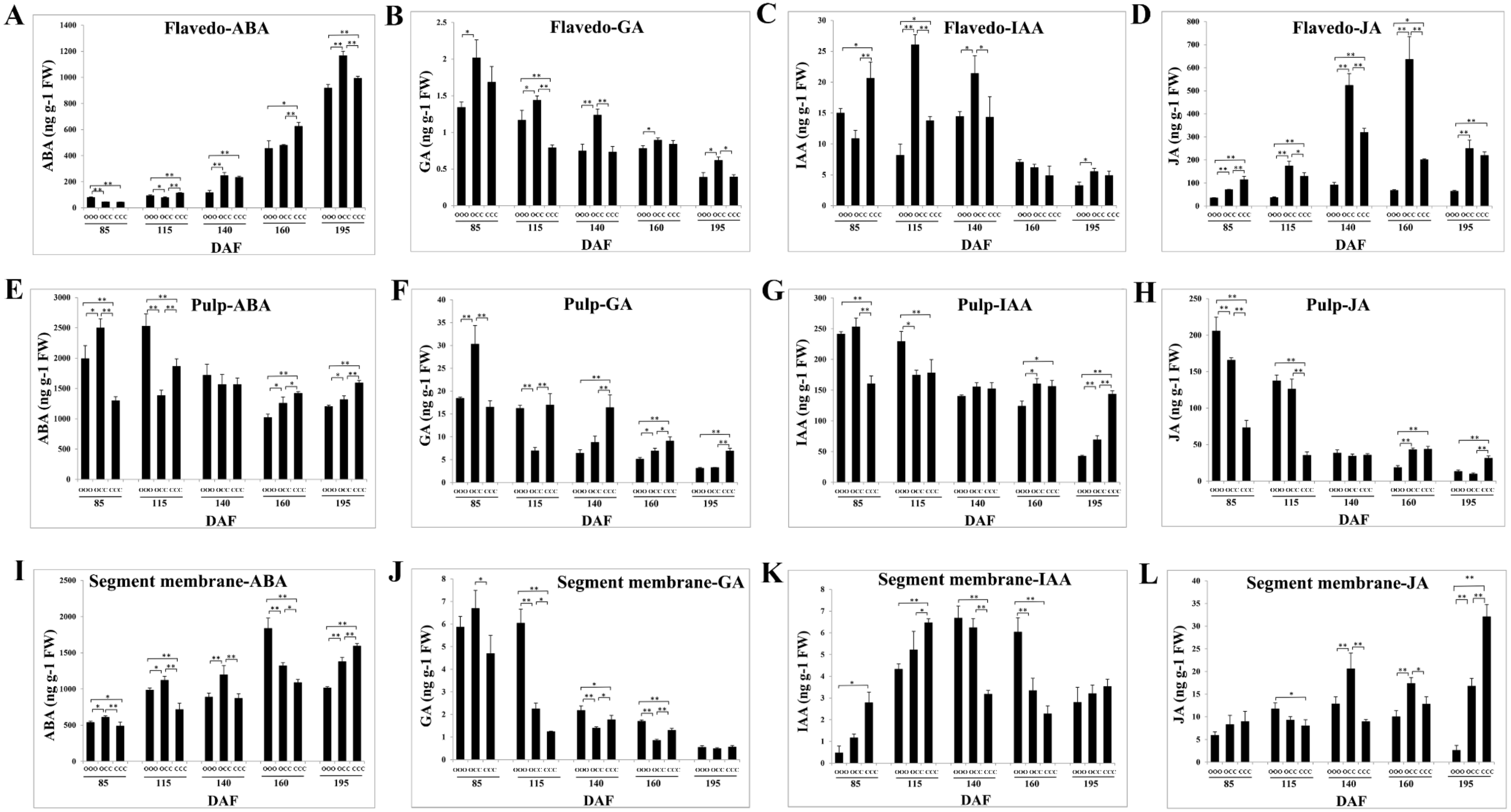

Supplementary Fig. S3 The phytohormone content in different tissues of ‘Owari’ Satsuma mandarin (OOO), 'Hongrou Huyou' (OCC), and ‘Changshan Huyou’ (CCC) during fruit development. (A-D) Changes in the ABA, GA, IAA, and JA, respectively, in flavedo of OCC and its donors at five different development stages. (E-H) Changes in the ABA, GA, IAA, and JA, respectively, in pulp of OCC and its donors at five different development stages. (I-L) Changes in the ABA, GA, IAA, and JA, respectively, in segment membrane of OCC and its donors at five different development stages. Means ± SD from three biological replicates are shown. Asterisks indicate statistically significant differences compared with Rm (Student's *t*-test *P*-value, **P* < 0.05, ***P* < 0.01).

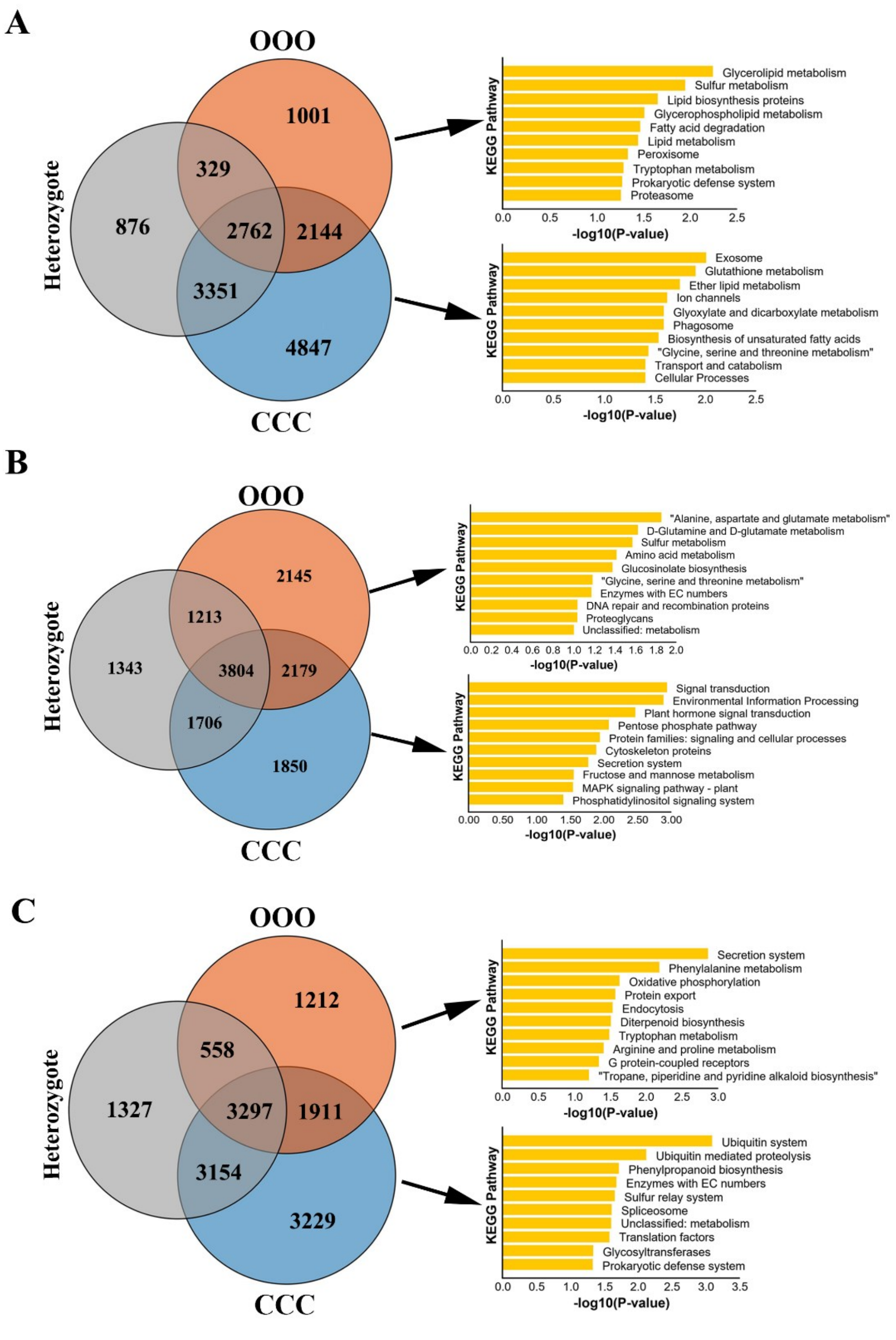

Supplementary Fig. S4 KEGG pathway enrichment statistics of layer-specific expression genes in flavedo, pulp, and segment membrane of OCC at the 195 DAF. (A) Venn diagram representation of the number of layer-specific expression genes in flavedo of OCC at the 195 DAF (left). KEGG pathway enrichment statistics of layer-specific expression genes in flavedo of OCC at the 195 DAF (right). (B) Venn diagram representation of the number of the expressed genes from different layers in pulp of OCC at the 195 DAF (left). KEGG pathway enrichment statistics of the expressed genes from different layers in pulp of OCC at the 195 DAF (right). (C) Venn diagram representation of the number of layer-specific expression genes in the segment membrane of OCC at the 195 DAF (left). KEGG pathway enrichment statistics of layer-specific expression genes in the segment membrane of OCC at the 195 DAF (right). Abbreviations are as follows: OOO, ‘Owari’ satsuma mandarin; OCC, ‘Hongrou Huyou’; CCC, ‘Changshan Huyou’.

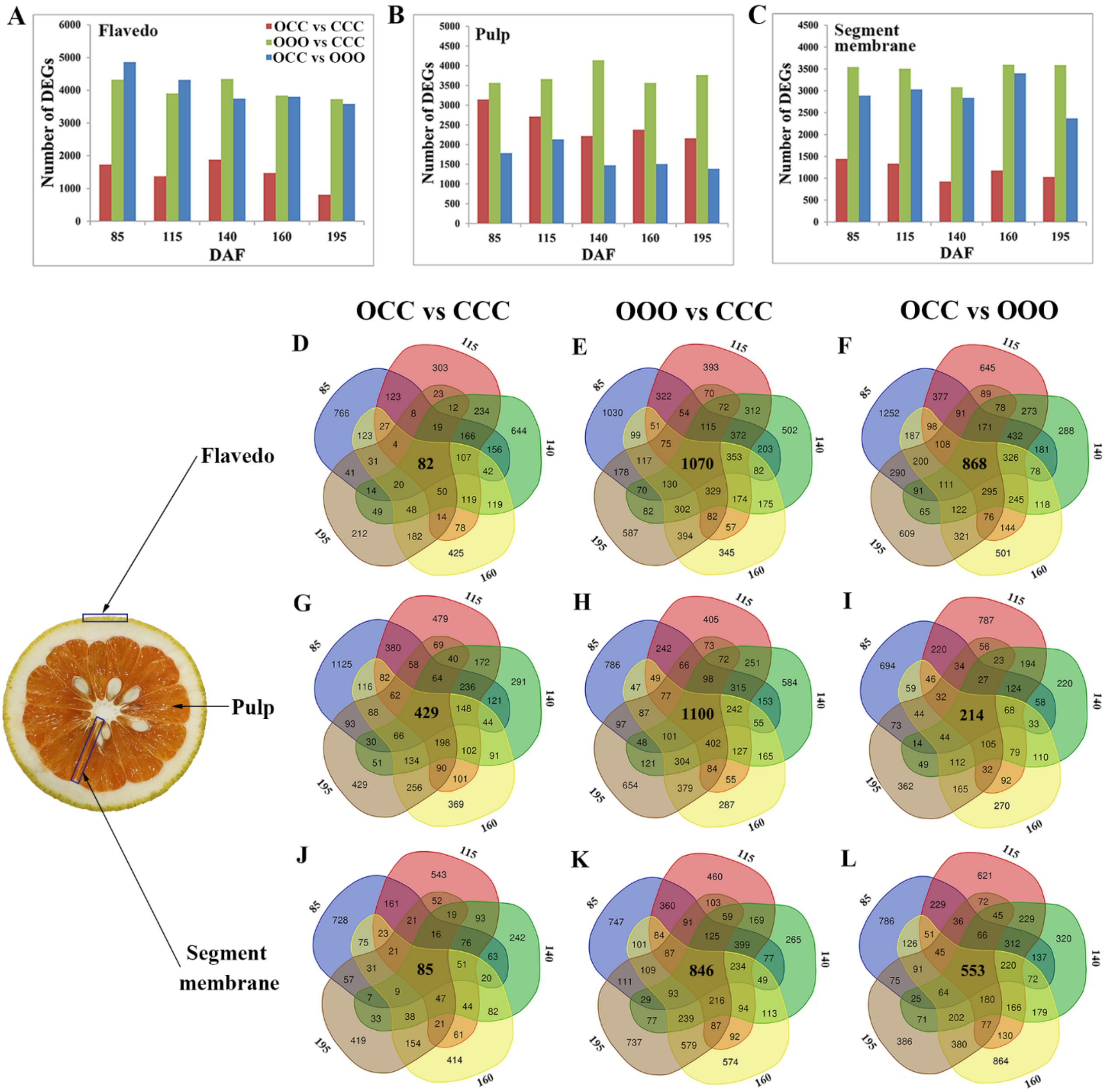

Supplementary Fig. S5 The number of differentially expressed genes (DEGs) identified by RNA-seq analysis among various fruit tissues of 'Owari' Satsuma mandarin (OOO), 'Hongrou Huyou' (OCC), and 'Changshan Huyou' (CCC) at five developmental stages. (A-C) The number of DEGs in pairwise comparison among the three species in flavedo, pulp, and segment membrane, respectively. (D-F) Venn diagram representation of the number of DEGs in OCC vs CCC, OOO vs CCC, and OCC vs OOO in flavedo, respectively. (G-I) Venn diagram representation of the number of DEGs in OCC vs CCC, OOO vs CCC, and OCC vs OOO in pulp, respectively. (J-L) Venn diagram representation of the number of DEGs in OCC vs CCC, OOO vs CCC, and OCC vs OOO in segment membrane, respectively.

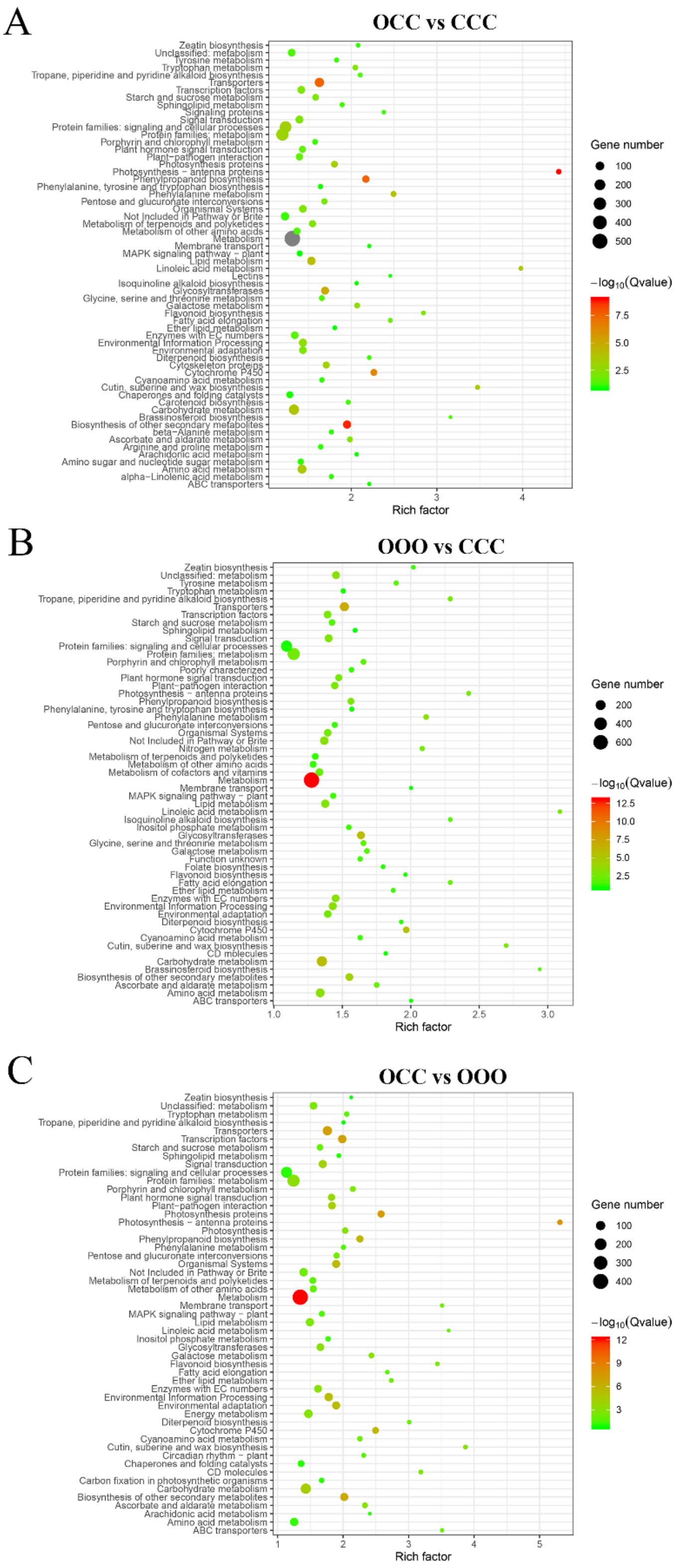

Supplementary Fig. S6 Scatterplot of KEGG pathway enrichment statistics in pulp of ‘Owari’ Satsuma mandarin (OOO), 'Hongrou Huyou' (OCC), and ‘Changshan Huyou’ (CCC) during different developmental stages. (A) KEGG pathway enrichment of DEGs between OCC and CCC. (B) KEGG pathway enrichment of DEGs between OOO and CCC. (C) KEGG pathway enrichment of DEGs between OCC and OOO.

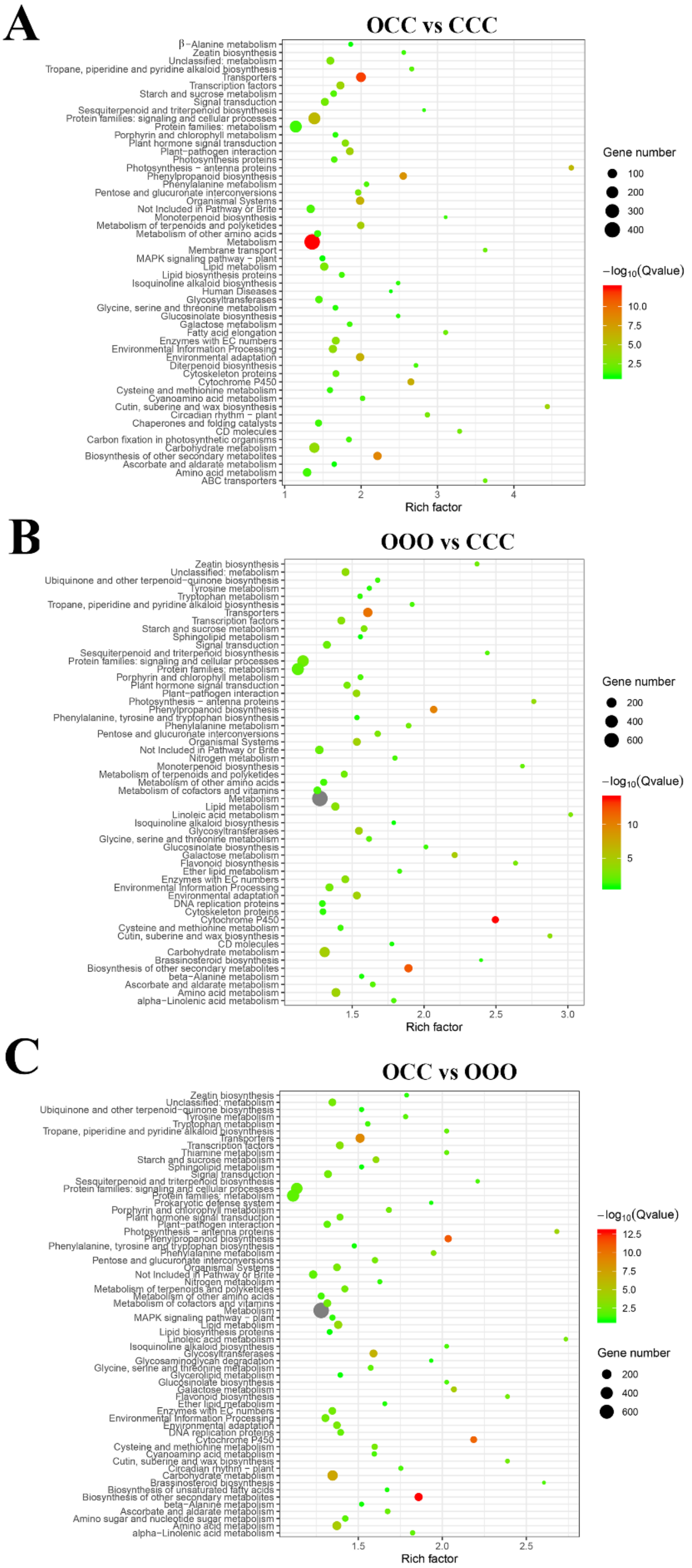

Supplementary Fig. S7 Scatterplot of KEGG pathway enrichment statistics in flavedo of ‘Owari’ Satsuma mandarin (OOO), 'Hongrou Huyou' (OCC), and ‘Changshan Huyou’ (CCC) during different developmental stages. (A) KEGG pathway enrichment of DEGs between OCC and CCC. (B) KEGG pathway enrichment of DEGs between OOO and CCC. (C) KEGG pathway enrichment of DEGs between OCC and OOO.

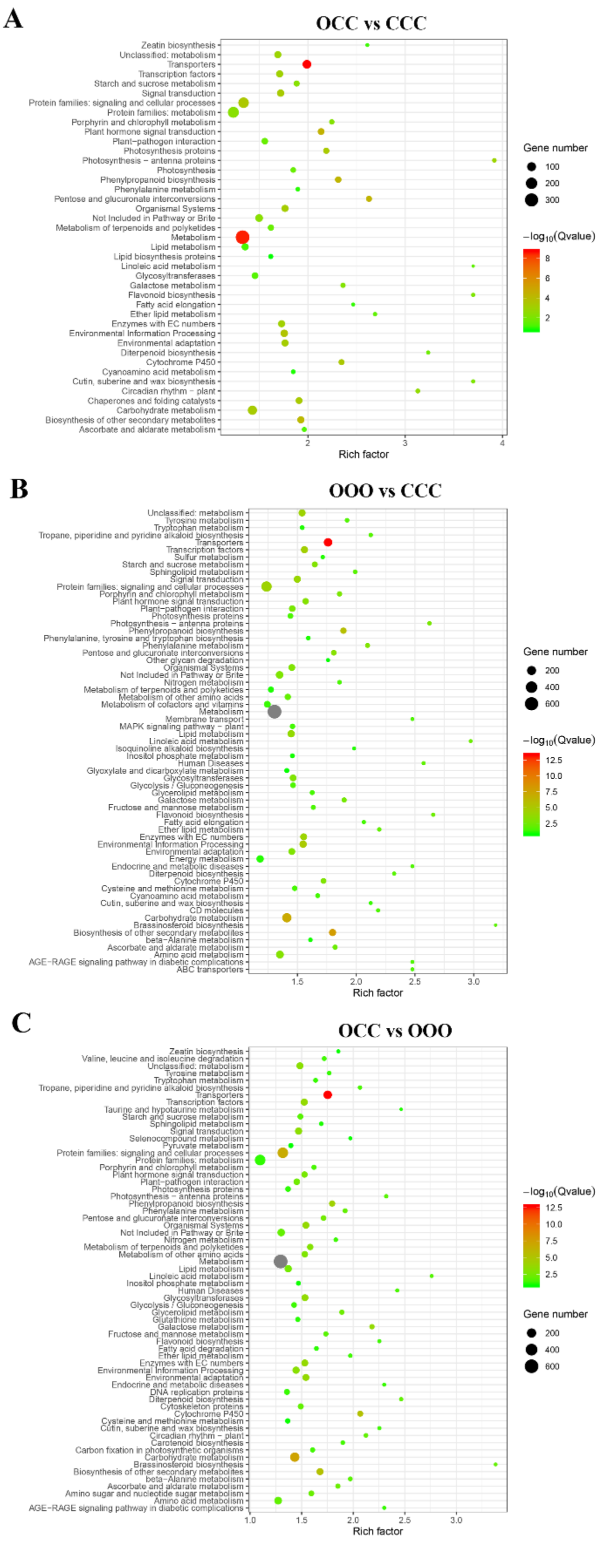

Supplementary Fig. S8 Scatterplot of KEGG pathway enrichment statistics in segment membrane of 'Owari' Satsuma mandarin (OOO), 'Hongrou Huyou' (OCC), and 'Changshan Huyou' (CCC) during different developmental stages. (A) KEGG pathway enrichment of DEGs between OCC and CCC. (B) KEGG pathway enrichment of DEGs between OOO and CCC. (C) KEGG pathway enrichment of DEGs between OCC and OOO.

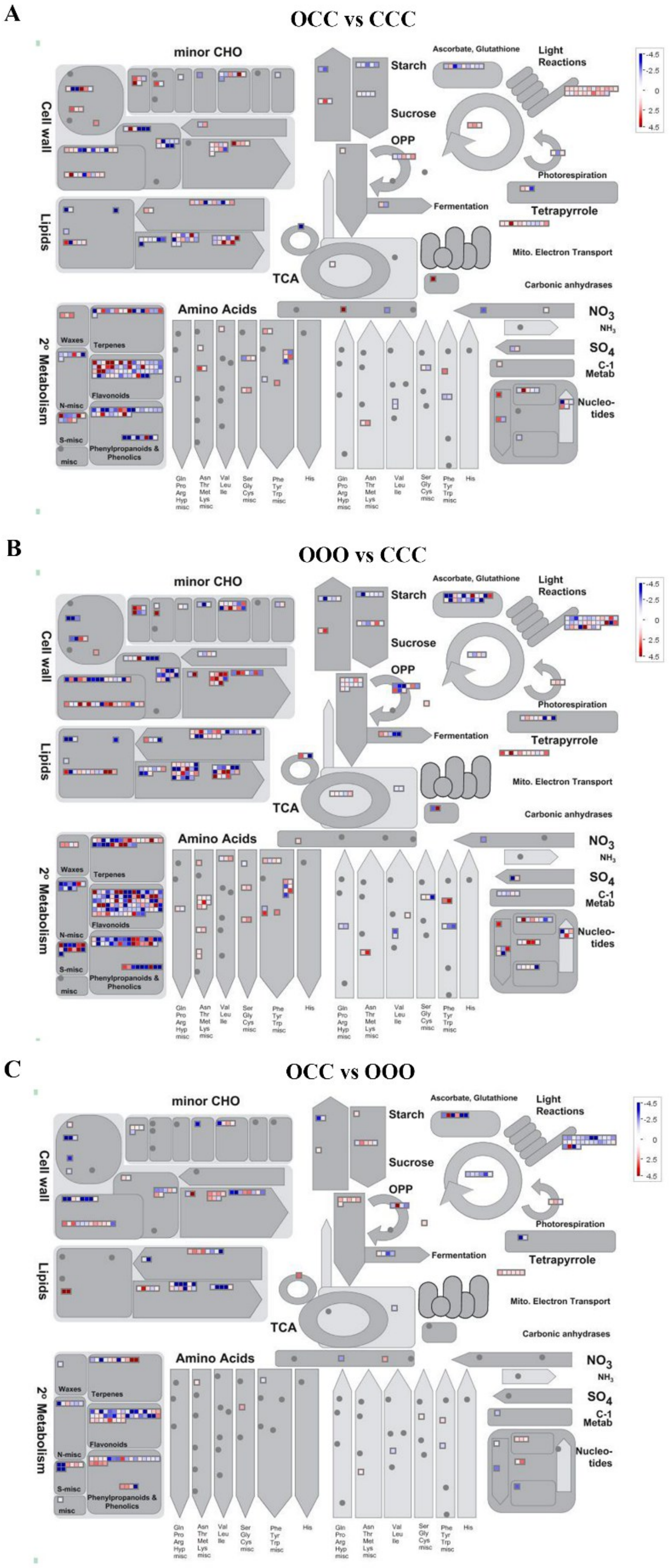

Supplementary Fig. S9 Mapman representations of DEGs in pulp of 'Owari' Satsuma mandarin (OOO), 'Hongrou Huyou' (OCC), and 'Changshan Huyou' (CCC) at 160 DAF stage. (A) Mapman analysis of DEGs between OCC and CCC. (B) Mapman analysis of DEGs between OOO and CCC. (C) Mapman analysis of DEGs between OCC and OOO.

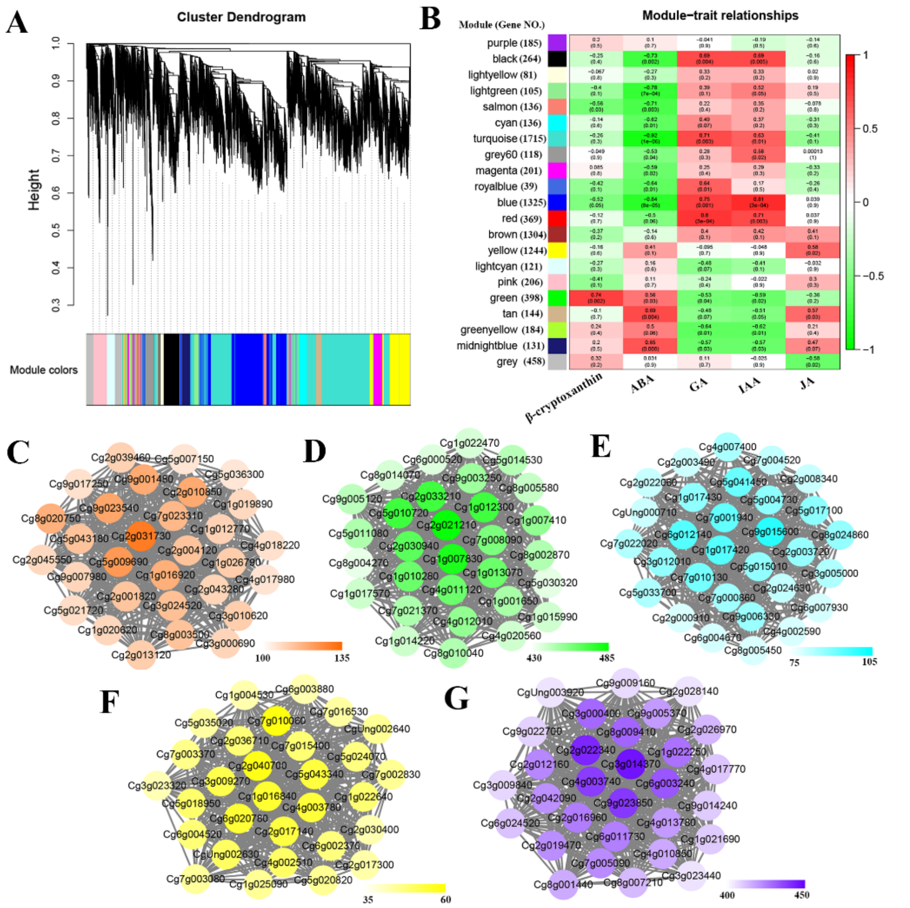

Supplementary Fig. S10 Weighted gene co-expression network analysis (WGCNA) of differentially expressed genes (DEGs) identified in flavedo of 'Owari' Satsuma mandarin (OOO), 'Hongrou Huyou' (OCC), and 'Changshan Huyou' (CCC) during different developmental stages. (A) Hierarchical cluster tree of the gene network. Each of the 8864 DEGs is represented by a leaf in the tree. The major tree branches constitute 21 modules labeled in different colors. Note that the module 'Grey' is for unassigned genes. (B) Module-carotenoid, ABA, GA, IAA, and JA correlations and the corresponding P-values (in parenthesis). Each row corresponds to a module. The left panel shows the 21 modules and the number of module member genes. Each column corresponds to a specific metabolite (carotenoid, ABA, GA, IAA, and JA). The color scale on the right shows module-trait correlation from -1 (green) to 1 (red). (C) Cytoscape representation of the top 30 co-expressed genes with an edge weight ≥0.10 in module 'Green', which is related to carotenoid. The edge number of the genes ranges from 100 to 135 (color-coded by the scale on the bottom right from white through orange). Member gene IDs were given. (D) Cytoscape representation of the top 30 co-expressed genes with an edge weight ≥0.10 in module 'Blue', which is related to IAA. The edge number of the genes ranges from 430 to 485 (color-coded by the scale on the bottom right from white through green). Member gene IDs are given. (E) Cytoscape representation of the top 30 co-expressed genes with an edge weight ≥0.10 in module 'Red', which is related to GA. The edge number of the genes ranges from 75 to 105 (color-coded by the scale on the bottom right from white through blue). Member gene IDs are given. (F) Cytoscape representation of the top 30 co-expressed genes with an edge weight ≥0.10 in module 'Tan', which is related to ABA. The edge number of the genes ranges from 35 to 60 (color-coded by the scale on the bottom right from white through yellow). Member gene IDs are given. (G) Cytoscape representation of the top 30 co-expressed genes with an edge weight ≥0.10 in module 'Yellow', which is related to JA. The edge number of the genes ranges from 400 to 450 (color-coded by the scale on the bottom right from white through purple). Member gene IDs are given.

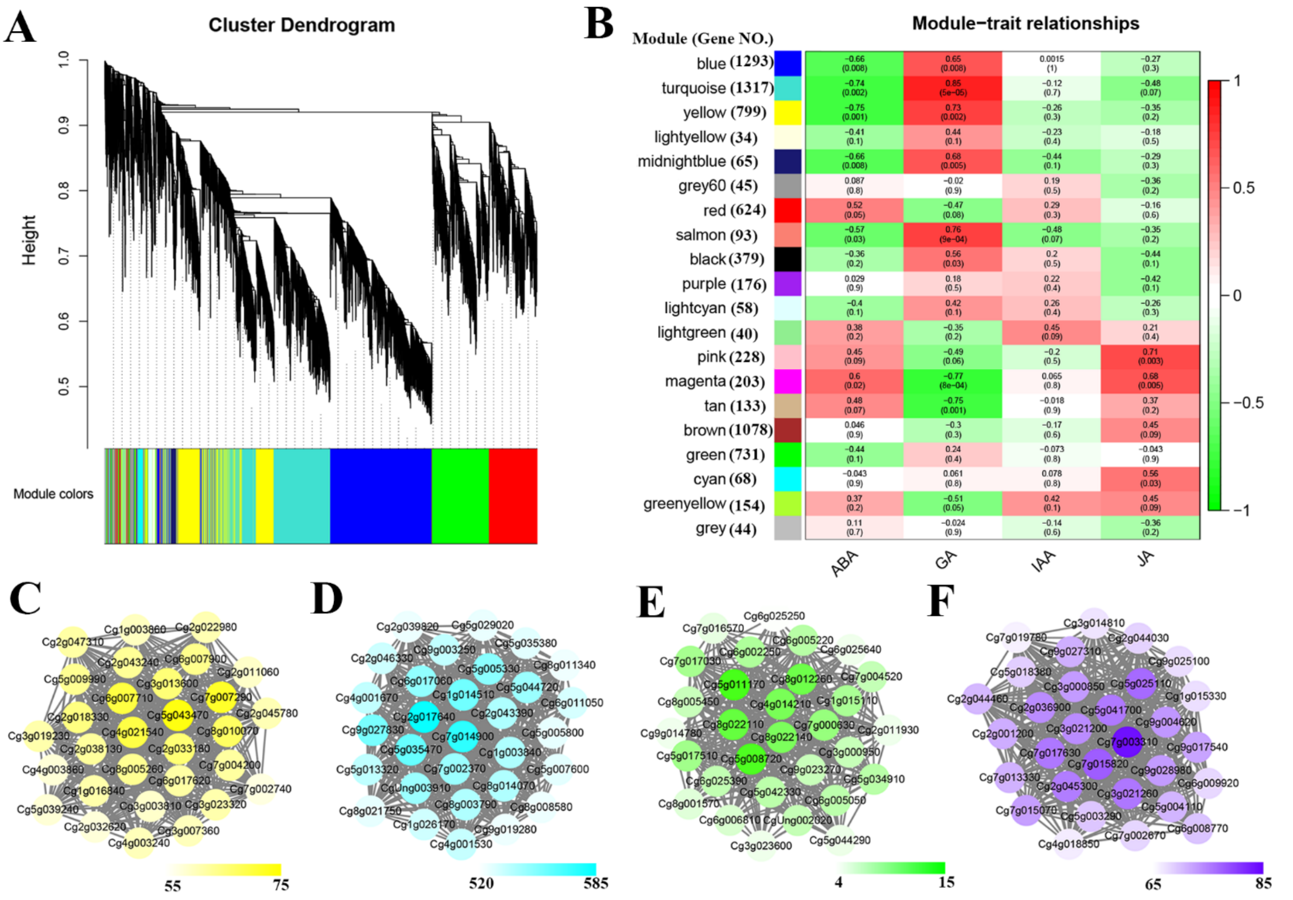

Supplementary Fig. S11 Weighted gene co-expression network analysis (WGCNA) of differentially expressed genes (DEGs) identified in segment membrane of ‘Owari’ Satsuma mandarin (OOO), 'Hongrou Huyou' (OCC), and ‘Changshan Huyou’ (CCC) during different developmental stages. (A) Hierarchical cluster tree of the gene network. Each of the 7562 DEGs is represented by a leaf in the tree. The major tree branches constitute 20 modules labeled in different colors. Note that the module 'Grey' is for unassigned genes. (B) Module- ABA, GA, IAA, and JA correlations and the corresponding P-values (in parenthesis). Each row corresponds to a module. The left panel shows the 20 modules and the number of module member genes. Each column corresponds to a specific metabolite (ABA, GA, IAA, and JA). The color scale on the right shows module-trait correlation from -1 (green) to 1 (red). (C) Cytoscape representation of the top 30 co-expressed genes with an edge weight ≥0.10 in module ‘Magenta’, which is related to ABA. The edge number of the genes ranges from 55 to 75 (color-coded by the scale on the bottom right from white through yellow). Member gene IDs are given. (D) Cytoscape representation of the top 30 co-expressed genes with an edge weight ≥0.10 in module ‘Turquoise’, which is related to GA. The edge number of the genes ranges from 520 to 585 (color-coded by the scale on the bottom right from white through blue). Member gene IDs are given. (E) Cytoscape representation of the top 30 co-expressed genes with an edge weight ≥0.10 in module ‘Lightgreen’, which is related to IAA. The edge number of the genes ranges from 4 to 15 (color-coded by the scale on the bottom right from white through green). Member gene IDs are given. (F) Cytoscape representation of the top 30 co-expressed genes with an edge weight ≥0.10 in module ‘Pink’, which is related to JA. The edge number of the genes ranges from 65 to 85 (color-coded by the scale on the bottom right from white through purple). Member gene IDs are given.

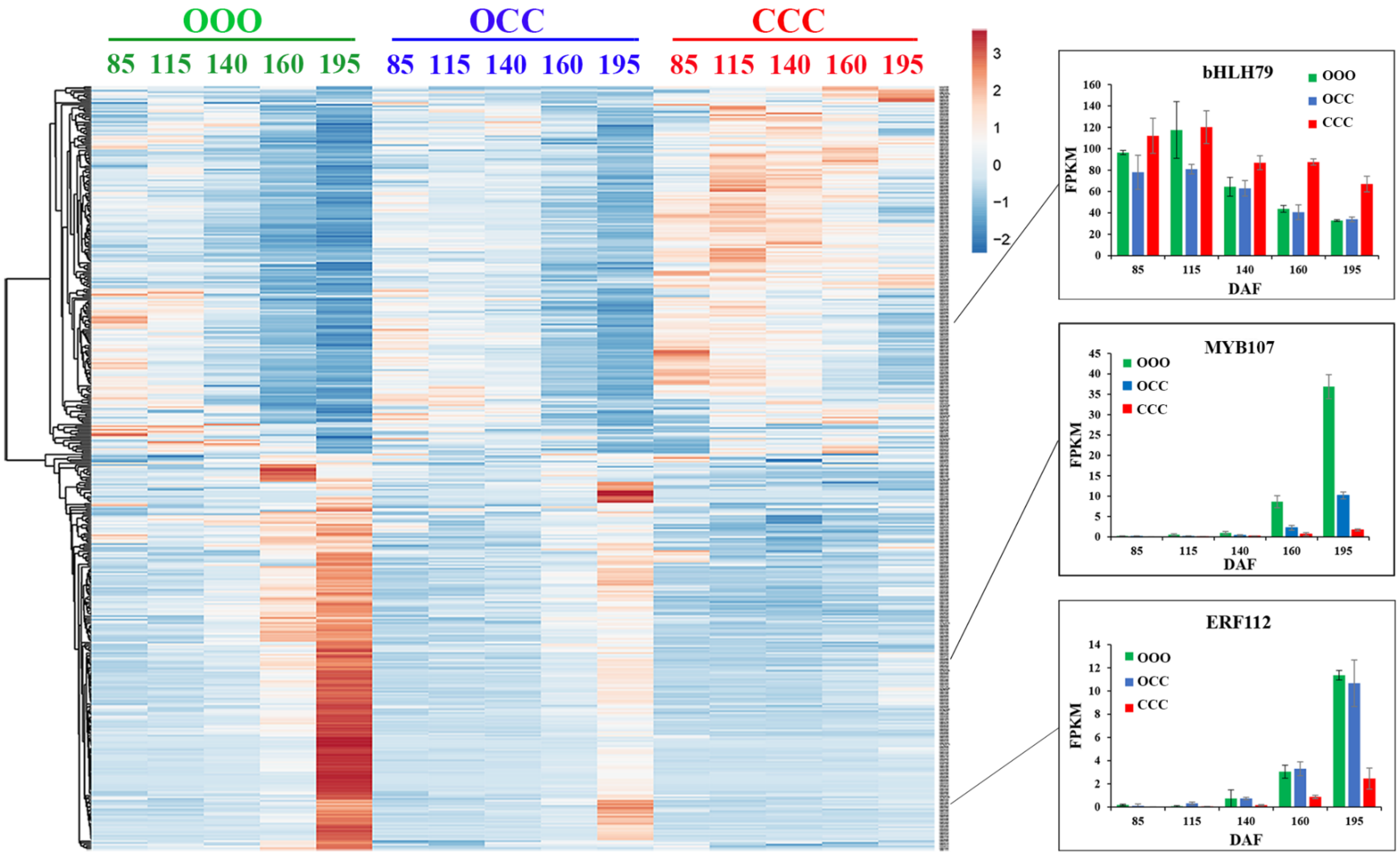

Supplementary Fig. S12 Heatmap of expression profiling of DEGs related to carotenoid accumulation in the WGCNA analysis of pulp. This analysis illustrates the expression profiles of DEGs related to carotenoid in the WGCNA analysis of pulp (left). FPKM of the candidate genes *bHLH79*, *MYB*, and *ERF112* (right). Abbreviations are as follows: OOO, 'Owari' satsuma mandarin; OCC, 'Hongrou Huyou'; CCC, 'Changshan Huyou'. Values are expressed as mean ± SD (n = 3).

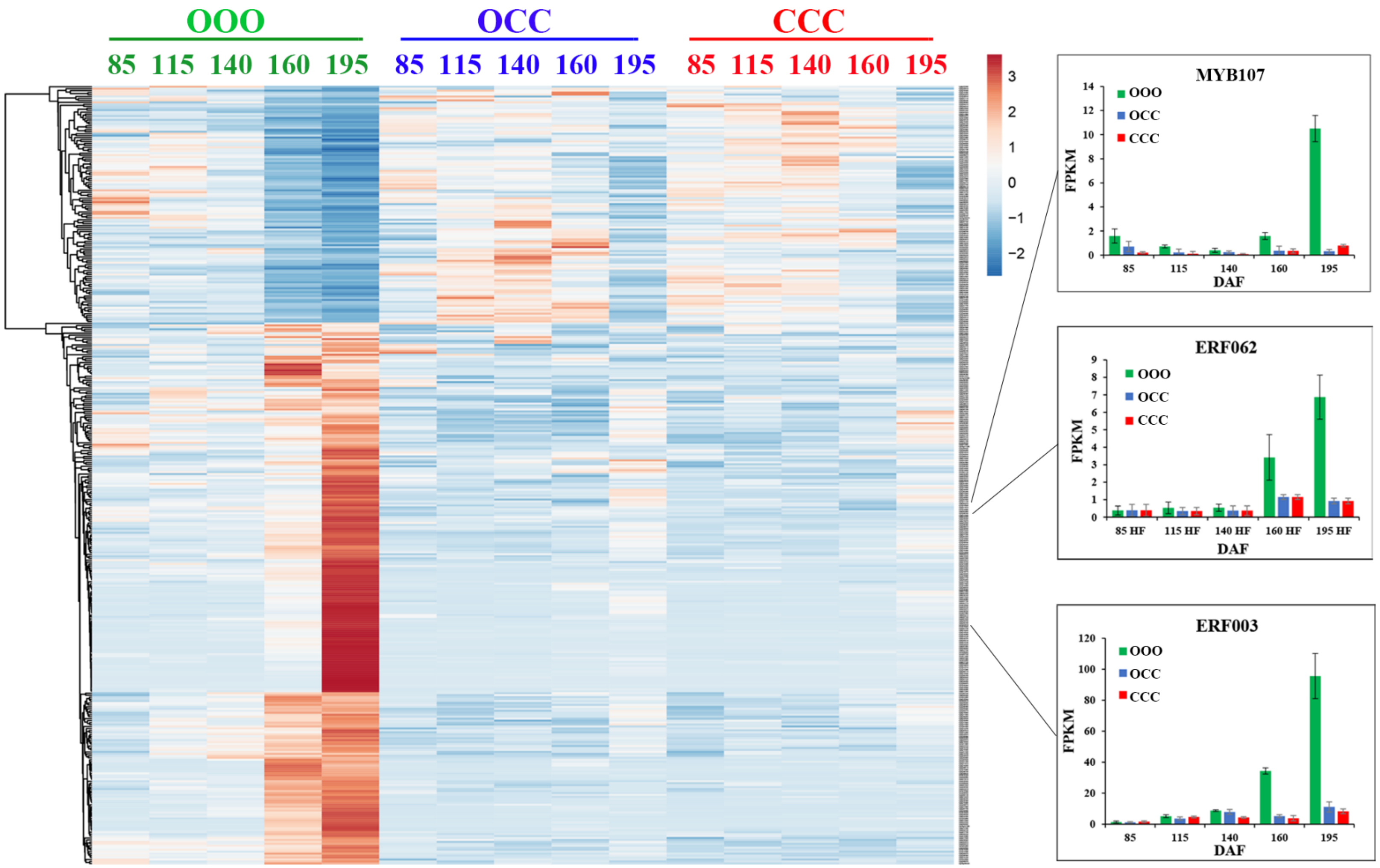

Supplementary Fig. S13 Heatmap of expression profiling of DEGs related to carotenoid accumulation in the WGCNA analysis of flavedo. This analysis illustrates the expression profiles of DEGs related to carotenoid in the WGCNA analysis of flavedo (left). FPKM of the candidate genes *MYB*, *ERF062,* and *ERF003* (right). Abbreviations are as follows: OOO, 'Owari' satsuma mandarin; OCC, 'Hongrou Huyou'; CCC, 'Changshan Huyou'. Values are expressed as mean ± SD (*n* = 3).

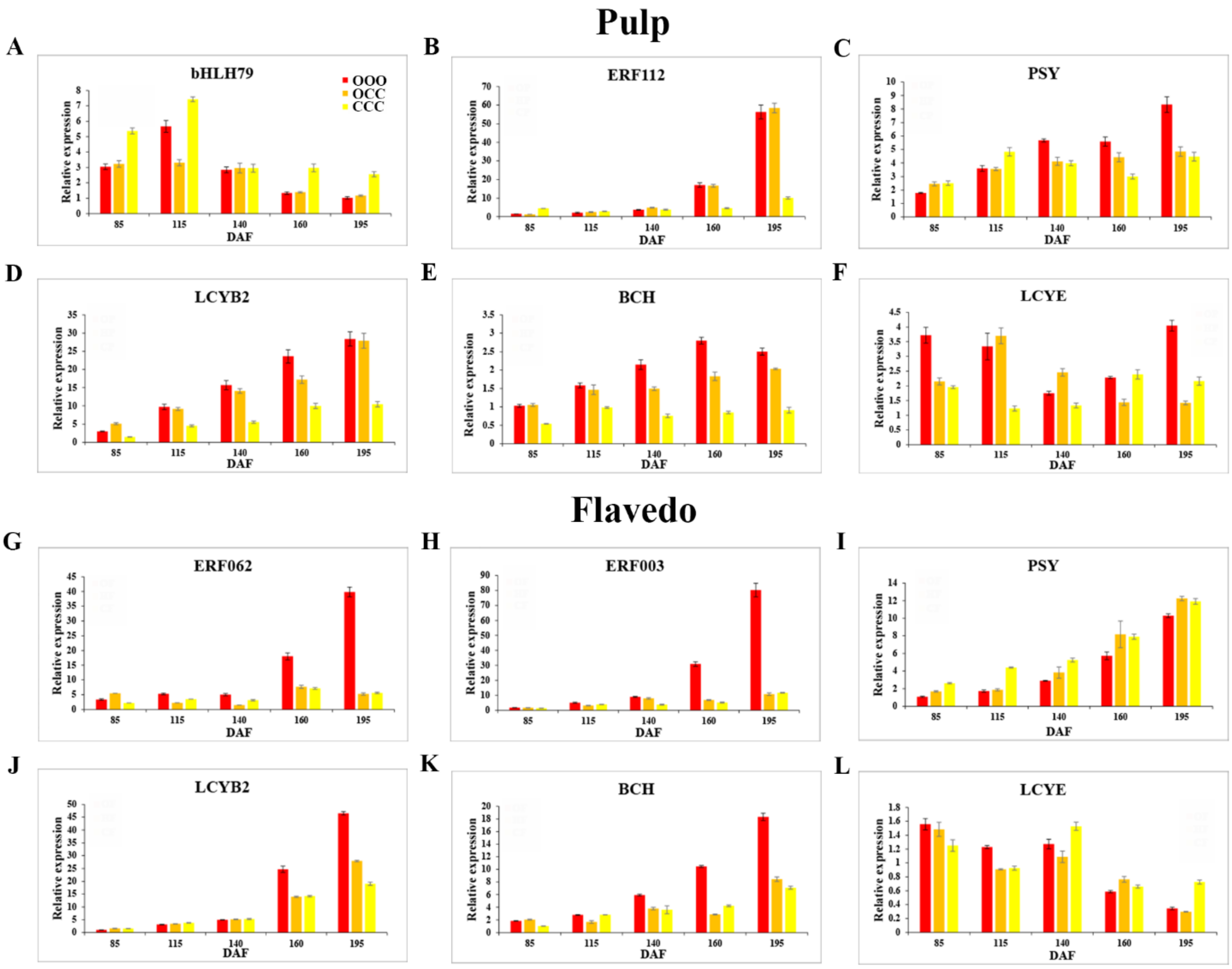

Supplementary Fig. S14 Expression profiles of representative genes measured by RNA-seq. The expression of the representative genes (*MYB107*, *bHLH79*, *ERF112*, *ERF062*, *ERF003*, *PSY*, *LCYB2*, *BCH*, and *LCYE*) from the RNA-seq analysis was analyzed in pulp or flavedo using qRT-PCR. Abbreviations are as follows: OOO, ‘Owari’ satsuma mandarin; OCC, ‘Hongrou Huyou’; CCC, ‘Changshan Huyou’. Values are expressed as mean ± SD (n = 3).

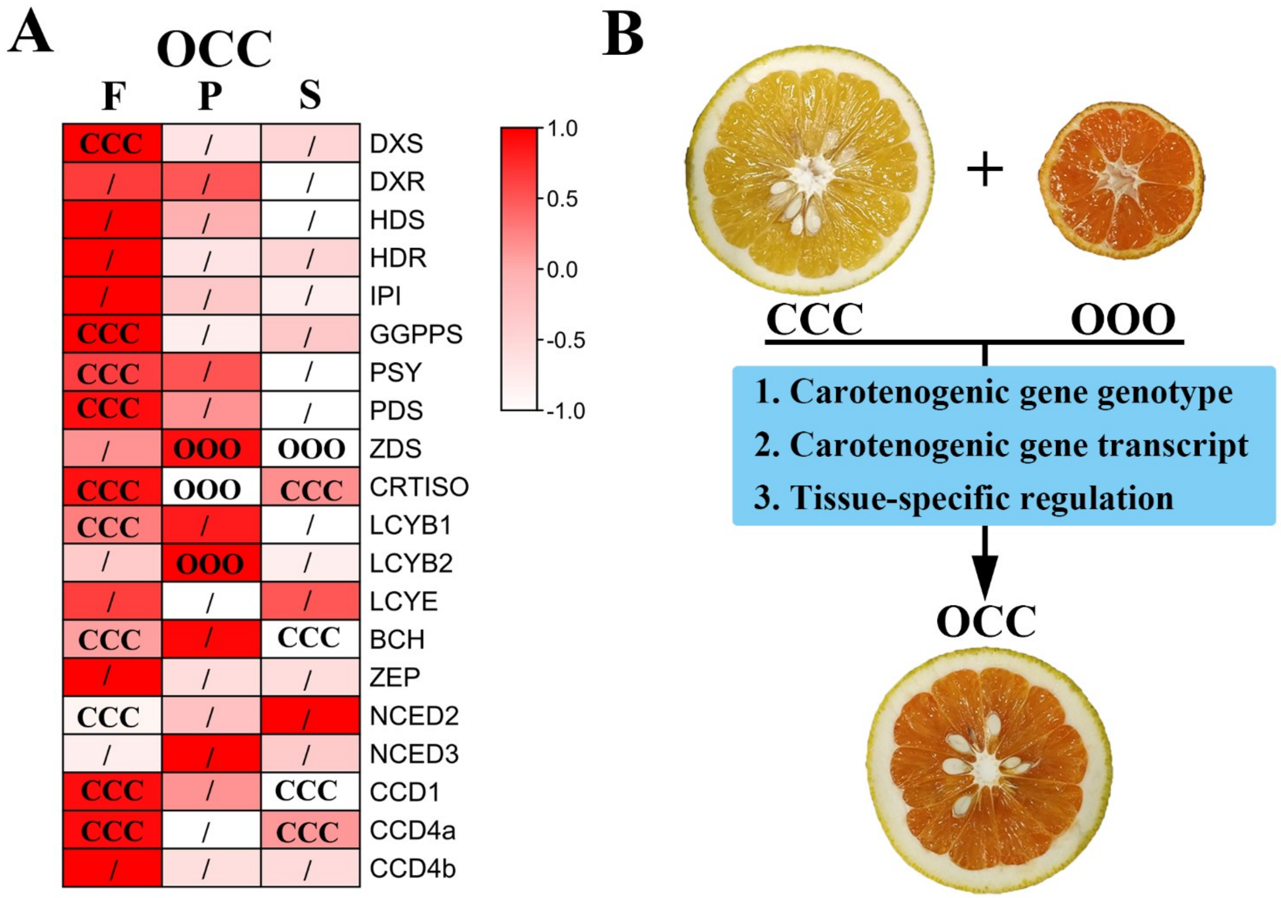

Supplementary Fig. S15 The tissue-specific expression patterns of carotenogenic genes identified in OCC at the 195 DAF and the model of β-cryptoxanthin accumulation in OCC. (A) Heatmap analysis of the tissue-specific expression of carotenogenic genes in OCC, donor origin of carotenogenic genes in OCC is marked. Abbreviations and symbols are as follows: F, flavedo; P, pulp; S, segment membrane; OOO, 'Owari' satsuma mandarin; OCC, 'Hongrou Huyou'; CCC, 'Changshan Huyou'; /, heterozygote. (B) The model of β-cryptoxanthin accumulation in OCC. β-cryptoxanthin accumulation is controlled by carotenogenic gene genotype and transcript, as well as tissue-specific regulation.

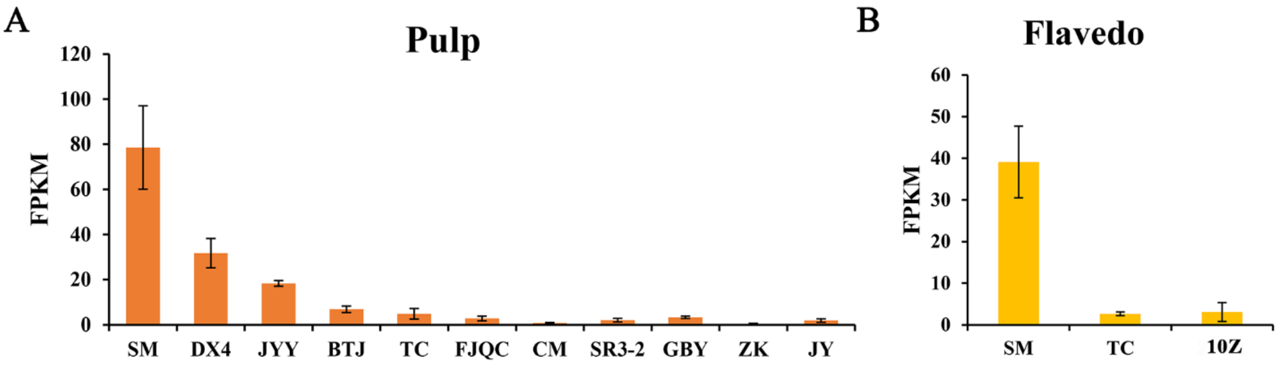

Supplementary Fig. S16 Expression profiles of *MYB107* among various citrus species measured by RNA-seq. Fragments per kilobase million (FPKM) of *MYB107* in the pulp (A) or flavedo (B) among various citrus species with diversity in β-cryptoxanthin accumulation. Abbreviations are referred to Table S15. RNA-seq data come from the Citrus Pan-Genome to Breeding Database (CPBD).
